# A Simplified SARS-CoV-2 Pseudovirus Neutralization Assay

**DOI:** 10.3390/vaccines9040389

**Published:** 2021-04-15

**Authors:** Gaetano Donofrio, Valentina Franceschi, Francesca Macchi, Luca Russo, Anna Rocci, Valentina Marchica, Federica Costa, Nicola Giuliani, Carlo Ferrari, Gabriele Missale

**Affiliations:** 1 Department of Medical-Veterinary Science, University of Parma, 43126 Parma, Italy; valentina.franceschi@unipr.it (V.F.); francesca.macchi@unipr.it (F.M.); luca.russo@unipr.it (L.R.); 2 Unit of Angiology and Internal Medicine, Azienda Ospedaliero-Universitaria di Parma, 43126 Parma, Italy; arocci@ao.pr.it; 3 Department of Medicine and Surgery, University of Parma, 43126 Parma, Italy; valentina.marchica@unipr.it (V.M.); federica.costa@unipr.it (F.C.); nicola.giuliani@unipr.it (N.G.); carlo.ferrari@unipr.it (C.F.); gabriele.missale@unipr.it (G.M.); 4 Unit of Hematology, Azienda Ospedaliero-Universitaria di Parma, 43126 Parma, Italy; 5 Laboratory of Viral Immunopathology, Unit of Infectious Diseases and Hepatology, Azienda Ospedaliero-Universitaria di Parma, 43126 Parma, Italy

**Keywords:** SARS-CoV-2, COVID-19, neutralizing antibody, pseudovirus, neutralization assay

## Abstract

COVID-19 is an ongoing pandemic caused by the highly infectious coronavirus SARS-CoV-2 that is engaging worldwide scientific research to find a timely and effective eradication strategy. Great efforts have been put into anti-COVID-19 vaccine generation in an effort to protect the world population and block SARS-CoV-2 spread. To validate the protective efficacy of the vaccination campaign and effectively control the pandemic, it is necessary to quantify the induction of neutralizing antibodies by vaccination, as they have been established to be a correlate of protection. In this work, a SARS-CoV-2 pseudovirus neutralization assay, based on a replication-incompetent lentivirus expressing an adapted form of CoV-2 S protein and an ACE2/TMPRSS2 stably expressing cell line, has been minimized in terms of protocol steps without loss of accuracy. The goal of the present simplified neutralization system is to improve SARS-CoV-2 vaccination campaign by means of an easy and accessible approach to be performed in any medical laboratory, maintaining the sensitivity and quantitative reliability of classical serum neutralization assays. Further, this assay can be easily adapted to different coronavirus variants by simply modifying the pseudotyping vector.

## 1. Introduction

Coronaviruses, belonging to the family Coronaviridae in the order Nidovirales, are positive-strand RNA viruses with a genome length of between 26 and 32 kbp. Several mammalian and avian species can be infected by coronaviruses, which most of the time cause respiratory and/or intestinal disease [1]. Human coronaviruses (HCoVs), such as HCoV-229E, HCoV-OC43, HCoV-NL63, and HKU1 (human coronavirus HCov-HKU1), have long been recognized as major causes of the common cold and are endemic in the human population. Two recent HCoVs, severe acute respiratory syndrome coronavirus (SARS-CoV) and Middle East respiratory syndrome coronavirus (MERS-CoV), emerged in 2002 and 2012, respectively, causing life-threatening disease in humans. A previously unknown coronavirus, named SARS-CoV-2 (CoV-2), was discovered in December 2019 in Wuhan, China, and has been responsible for a pandemic infection, known as coronavirus disease 19 (COVID-19), causing a large number of deaths people worldwide [1]. Although great research effort has been made, COVID-19 remains a complex disease showing pathogenetic mechanisms and clinical heterogeneous features that are difficult to understand. A variety of approaches have been employed to develop prophylactic and therapeutic measures, including whole inactivated vaccines, subunit vaccines, RNA-based vaccines, viral vectored vaccines [2,3], monoclonal neutralizing antibodies, and fusion inhibitors, most of which were designed to target the CoV-2 spike glycoprotein (S) [4,5,6]. CoV-2 S, forming homotrimer structures on the viral surface, mediates virus entry to the host cell. S mature structure is formed by S1 and S2 functional subunits: S1 interacts with the angiotensin-converting enzyme 2 (ACE2) cellular receptor, while S2 mediates the viral envelope fusion with the host cell membrane [7,8,9]. During S1–ACE2 interaction, the host cell surface transmembrane serine protease 2 (TMPRSS2), located next to ACE2 receptor, cleaves the S2 subunit at the S2′ amino-terminal portion (815↓816aa; SKR↓SFI), inducing the exposure of fusion peptide hydrophobic domains and the subsequent viral envelope fusion with the host cell membrane [7,8,9]. Due to its high infectivity and pathogenicity, CoV-2 needs to be handled in biosafety level 3 (BSL-3) specific facilities (https://www.cdc.gov/coronavirus/2019-ncov/lab/lab-biosafety-guidelines.html, accessed on 5 June 2020) [10], which limits the development of antiviral measures as well as basic and applied studies on the interaction between host cells and CoV-2 and viral attachment and entry mediated by the S protein. To avoid dealing with infectious CoV-2, several safe, biosafety level 2 (BSL2) pseudovirus-based systems have been developed, mainly based on vesicular stomatitis virus (VSV) [11] or retrovirus (RV) [12,13] vector pseudotyped with CoV-2 S. Although both of them have been shown to be sensitive and reliable, they suffer from being farraginous, time-consuming, and expensive in procedural terms. In the present work, a four-step simplified procedure of CoV-2 pseudovirus neutralization assay was established.

## 2. Material and Methods

### 2.1. Plasmids

ACE2-IRES-TMPRSS2-IRES-Puromycin tricistronic ORF was chemically synthesized and integrated into a lentiviral transfer vector to obtain pEF1α-ACE2/TMPRRS2/Puro (Figure S1 for details and full sequence). Similarly, S-ΔRS-HA ORF (Figure S2) and biscistronic turboGFP-IRES-Luc2 ORF were chemically synthesized and integrated into a lentiviral transfer vector to obtain pLV-CMV-(S-ΔRS-HA)-IRES-Puro-WPRE (Figure S3) and pLV-EF1α-(turboGFP-IRES-Luc2)-WPRE, respectively. p8.74 packaging, pREV and pMD2 pseudotyping, and pEGFP-C1 vectors were obtained from Addgene (https://www.addgene.org/, accessed on 3 May 2020).

### 2.2. Cells

Human Embryo Kidney (HEK) 293T (ATCC: CRL-11268) cells were cultured in Eagle’s Minimal Essential Medium (EMEM, Gibco; Thermo Fisher Scientific, Carlsbad, CA, USA) containing 1 mM of sodium pyruvate (Gibco, Thermo Fisher Scientific, Carlsbad, CA, USA), 2 mM of L-glutamine (Gibco), 100 IU/mL of penicillin (Gibco), 100 μg/mL of streptomycin (Sigma-Aldrich, Milano, Italy), and 0.25 μg/mL of amphotericin B (Gibco) (called complete EMEM) supplemented with 10% fetal bovine serum (FBS, Gibco) and were incubated at 37 °C and 5% CO_2_ in a humidified incubator.

Stably transfected HEK/S-ΔRS-HA/Puro (HEK/S) and HEK/ACE2/TMPRRS2/Puro cells were obtained by transfecting cells with pLV-CMV-(S-ΔRS-HA)-IRES-Puro-WPRE or pEF1α-ACE2/TMPRRS2/Puro vectors, respectively. Briefly, subconfluent HEK 293T cells were detached from a T75 (75 cm^2^ surface area) flask, counted, and electroporated with 20 µg of pLV-CMV-(S-ΔRS-HA)-IRES-Puro-WPRE or pEF1α-ACE2/TMPRRS2/Puro vectors in 600 µL of DMEM high glucose (Euroclone, S.p.A, Milan, Italy) without serum at 186 V and 1500 µF in Gene Pulser XCell (Biorad, Milano, Italy).

Electroporated cells were then transferred to new 25 cm^2^ flasks and fed with complete EMEM with 10% FBS. Twenty-four hours after the transfection, the medium was changed with fresh complete EMEM with 10% FBS complemented with 2 µg/mL of puromycin (Millipore Merck Life Science, Milano, Italy). Cells were kept in culture until resistant colonies appeared. Cells were split for more than 40 passages and tested for S or ACE2 expression.

### 2.3. Transient Transfection and Syncytia Formation

HEK 293T cells were transiently cotransfected with pLV-CMV-(S-ΔRS-HA)-IRES-Puro-WPRE, pEF1α-ACE2/TMPRRS2/Puro and pEGFP-C1 (Clontech, Takara BIO, Mountain View, CA, USA) vectors, with same molar ration (1:1:1), using polyethylenimine (PEI) transfection reagent (Polysciences, Inc. Warrington, PA, USA). Briefly, cells were seeded at 5 × 10^5^ cells/well in 6-well plates and incubated overnight at 37 °C and 5% CO_2_. Cells were then incubated for 6 h with a transfection mix (1 mL) containing 3 μg of plasmids DNA and PEI (ratio 1:2.5 DNA-PEI) in complete DMEM (Dulbecco’s Modified Essential Medium (DMEM, Gibco; Thermo Fisher Scientific, Carlsbad, CA, USA) high glucose (Euroclone) completed with 50 μg/mL of gentamicin (Merk, Darmstadt, Germany) without serum. After incubation, the transfection mix was replaced by fresh complete EMEM and incubated for 24 h at 37 °C and 5% CO_2_. HEK/ACE2/TMPRRS2/Puro cells were transiently cotransfected with pLV-CMV-(S-ΔRS-HA)-IRES-Puro-WPRE and pEGFP-C1 as before. Alternatively, HEK/ACE2/TMPRRS2/Puro cells were also cocultivated (1:2 ratio) with HEK 293T cells transiently transfected with pLV-CMV-(S-ΔRS-HA)-IRES-Puro-WPRE and pEGFP-C1. Twenty-four hours after cocultivation, syncytia were observed by inverted fluorescence microscope (Zeiss-Axiovert-S100), and pictures were acquired by digital camera (Zeiss-Axiocam-MRC). HEK/S-ΔRS-HA/Puro and HEK/ACE2/TMPRRS2/Puro cells were also cocultivated as before to generate syncytia.

### 2.4. Western Immunoblotting

Protein cell extracts were obtained from pLV-CMV-(S-ΔRS-HA)-IRES-Puro-WPRE, pEF1α-ACE2/TMPRRS2/Puro and pEGFP-C1 stably transfected HEK293T cells by adding 100 μL of cell extraction buffer (50 mM Tris-HCl, 150 mM NaCl, and 1% NP-40; pH 8). After BCA total protein quantification (Pierce BCA Protein Assay kit, Thermo Fisher Scientific, Carlsbad, CA, USA), cell extracts containing various amounts of total protein were electrophoresed through 10% SDS-PAGE. Proteins were then transferred to a nylon membrane by electroblotting, and the membrane was incubated with mouse monoclonal antibody anti-HA tag (G036, Abm Inc., Vancouver, BC, Canada) diluted 1:10,000. After washing, the membrane was incubated with a rabbit anti-mouse IgG secondary antibody labeled with horseradish peroxidase, diluted 1:15,000 (A9044, Sigma-Aldrich, Merk, Darmstadt, Germany). For ACE2 detection, the membrane was incubated with rabbit monoclonal antibody anti-humanACE2 (SN0754, Invitrogen, Thermo Fisher Scientific, Carlsbad, CA, USA) diluted 1:5000. After washing, the membrane was incubated with a goat anti-rabbit IgG secondary antibody labeled with horseradish peroxidase, diluted 1:15,000 (A0545, Sigma-Aldrich). Bands were visualized by enhanced chemiluminescence (Clarity Max Western ECL substrate, Bio-Rad, Hercules, CA, USA).

### 2.5. Hematoxylin and Eosin Staining

Flasks of cells containing syncytia were fixed with 4% paraformaldehyde in PBS and stained with hematoxylin and eosin standard method.

### 2.6. Pseudovirions Reconstitution

For reconstituting CoV-2 S pseudovirus, HEK 293T cells were transfected, in T175 cm^2^ flasks, with 25 μg of pLV-EF1α-(turboGFP-Luc2)-WPRE transfer vector, 15 μg of p8.74 packaging vector, 13 μg of pLV-CMV-(S-ΔRS-HA)-IRES-Puro-WPRE pseudotyping vector (although this is a transfer vector, it can work as a pseudotyping vector too), and 5 μg of pREV (58 μg of total DNA) and were diluted in 3 mL of complete DMEM (Euroclone) without serum and 145 μL of PEI (Polysciences, Inc., Warrington, PA, USA) (1 mg/mL in PBS) (ratio 1:2.5 DNA/PEI). After at least 15 min incubation at room temperature, 4× volumes of complete DMEM without serum were added, and the transfection solution was transferred to the cell monolayer. After 6 h of incubation at 37 °C and 5% CO_2_, in a humidified incubator, the transfection mixture was replaced with 25 mL of fresh complete EMEM supplemented with 10% FBS and incubated for 48 h at 37 °C and 5% CO_2_. The flask was then frozen–thawed at −80 °C; transfected cell supernatant (TCS) containing S pseudovirus was clarified via centrifugation at 3500 rpm for 5 min at 4 °C, filtered through a 0.45 μm filter (Millipore, Merk, Darmstadt, Germany), aliquoted, tittered by limited dilution, and stored at −80 °C.

ACE2/TMPRSS2 pseudovirus was prepared as described above by simply substituting pLV-CMV-(S-ΔRS-HA)-IRES-Puro-WPRE with pEF1α-ACE2/TMPRRS2/Puro; again, although this is a transfer vector, it can work as a pseudotyping vector too.

### 2.7. Serum Samples

Sera were collaboratively provided by the Unit of Infectious Diseases and Hepatology, University Hospital of Parma. The study was approved by the local ethical committee (Comitato Etico Area Vasta Emilia Nord (AVEN), Italy). All participants gave written informed consent to participate in the study.

### 2.8. Seroneutralization Assay

Twenty-five microliters of complete EMEM with 10% FBS was added to each well of a 96-well clear–flat-bottom white microplate with opaque walls (Greiner Bio-One), and 25 µL of each serum was added to the first line of wells (Figure S5). Twenty-five microliters of S pseudovirus preparation (described in Section 2.6 above) diluted in complete EMEM with 10% FBS (corresponding to ~10^4^ relative luciferase units (RLUs); ~3–5µL of the initial preparation) was added to each well and left to incubate at room temperature for 1.5 h. Final volume for each well reached 50 µL; therefore, the sera dilution was doubled, 1:4–1:8–1:16–1:32–1:64–1:128–1:256–1:512. Next, 50 µL of complete EMEM with 10% FBS, containing 10^4^ HEK/ACE2/TMPRRS2/Puro cells, was added to each well and left for 60 h at 37 °C and 5% CO_2_.

Plates were read by adding 25 µL of complete EMEM containing luciferin to each well just before the reading of the microplate with the luminometer (Victor, Perkin Elmer). A negative control was established without serum, which was substituted with complete medium. The RLUs were compared and normalized to those derived from wells where pseudovirus was added in the absence of sera (100%). Neutralization titer 50 (NT50) was expressed as the maximal dilution of the sera where the reduction of the signal is ≥50%. Worthy of note, the titer had to be multiplied by 40 because the initial volume of the sera tested was 0.025 mL and it had to be normalized to 1 mL.

## 3. Results and Discussion

### 3.1. Generation of a Sensitive Target Cell Line Simultaneously Expressing ACE2 and TMPRRS2

As ACE2 and TMPRSS2 are the main dependency factors for SARS-CoV-2 (CoV-2) attachment and penetration into the host cells [8], a cell line simultaneously expressing ACE2 and TMPRSS2 was generated. For this purpose, a tricistronic expression cassette was constructed. Human ACE2 ORF (GenBank accession number: NM_021804.3), provided with a strong Kozak’s sequence to the 5′ end; human TMPRSS2 ORF (GenBank accession number: NM_001135099.1); and the puromycin drug resistance gene ORF were in-tandem positioned and linked by two internal ribosomal entry sites (IRESs) (ACE2-IRES-TMPRSS2-IRES-Puromycin). This large tricistronic ORF was chemically synthesized and subcloned downstream to the human elongation factor 1 alpha promoter (EF1α), upstream of the SV40 polyA signal/site in a pUC plasmid backbone. Thus, pEF1α-ACE2/TMPRRS2/Puro was generated (Figure S1A). pEF1α-ACE2/TMPRRS2/Puro was electroporated in HEK293T cells, and stably transfected cells were obtained by selection with puromycin. As puromycin drug resistance gene ORF is the last to be translated from the mRNA transcribed from pEF1α-ACE2/TMPRRS2/Puro, the puromycin selection of resistant cells (HEK/ACE2/TMPRRS2/Puro) increases the probability of the majority of the cells expressing ACE2 and TMPRSS2. HEK/ACE2/TMPRRS2/Puro cells were kept for over 30 passages without loss of functionality/expression (Figure S1B). Noteworthily, HEK/ACE2/TMPRRS2/Puro cells do not need to be constantly maintained in culture, under puromycin selection: a large batch of them can be grown, aliquoted at a suitable concentration, stored at −80 °C or in liquid nitrogen, and successively used, when needed, by simply thawing an aliquot of them, without any loss of functionality.

### 3.2. Expression of SARS-CoV-2 Spike Glycoprotein in Mammalian Cells

CoV-2 S is a key factor for SARS-CoV-2 target cell infection [7,8,9]. Antibodies from COVID-19 convalescent patient sera can block in vitro and in vivo CoV-2 infectivity; thus, they are exploitable for CoV-2 infection diagnosis and for the evaluation of CoV-2 vaccination efficacy (the titer of neutralizing antibody present in a serum is a correlate of protection), as well as for preliminary test molecules able to interfere with S, ACE2, and TMPRRS2 interaction. Before attempting the generation of a lentivirus-based pseudovirus displaying CoV-2 S on its surface, it was indispensable to generate an appropriate expression cassette capable of driving an efficient expression of S, accommodated on the pseudovirus envelope surface. The cytoplasmic tails of some CoV S proteins contain an endoplasmic reticulum retrieval signal (ERRS) that can retrieve S proteins from the Golgi to the endoplasmic reticulum (ER). This process is thought to accumulate S proteins at the CoV budding site, the ER–Golgi intermediate compartment (ERGIC), and to facilitate S protein incorporation into virions [14,15]. In contrast to coronaviruses, lentiviruses assemble and bud at the cell surface [16]; therefore, truncation of the S-protein cytoplasmic tail may increase cell surface levels and/or enable incorporation by alleviating structural incompatibility of the S-protein cytoplasmic tail and lentivirus proteins.

**Figure 1:**
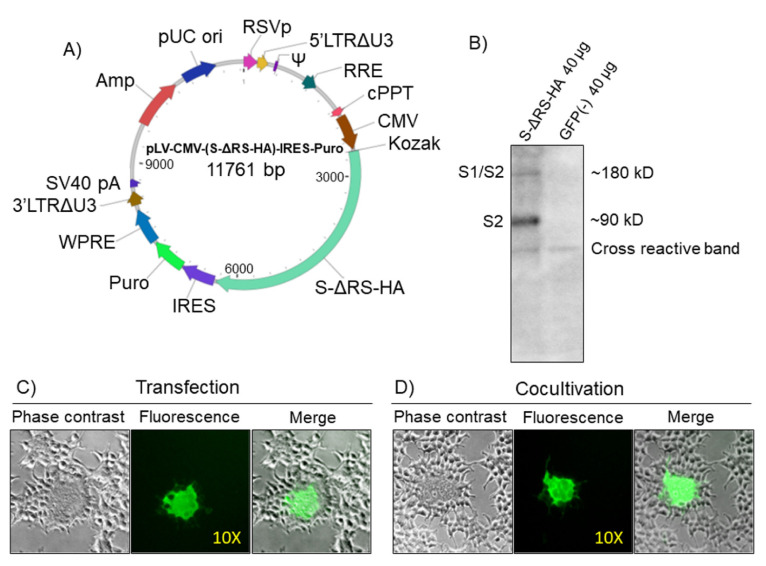
CoV-2 S expression in HEK293T cells. (**A**) Diagram (not to scale) of pLV-CMV-(S-ΔRS-HA)-IRES-Puro-WPRE transfer vector, where functional elements of the construct are indicated and highlighted with different colors. RSVp (Rous sarcoma virus enhancer/promoter), 5′LTRΔU3 (truncated HIV-1 5′ long terminal repeat), Ψ (HIV-1 packaging signal), RRE (HIV-1 REV response element), cPPT (central polypurine tract), CMV (human cytomegalovirus immediate early enhancer/promoter), Kozak (Kozak’s translation initiation sequence), S-ΔRS-HA (human codon usage adapted, HA-tagged and retention-signal-deleted CoV-2 S ORF), IRES (encephalomyocarditis virus internal ribosomal entry site), Puro (puromycin-resistant gene ORF), WPRE (woodchuck hepatitis virus posttranslational regulatory element), 3′LTRΔU3 (truncated HIV-1 3′ long terminal repeat; self-inactivation), SV40 early pA (simian virus 40 early polyadenylation signal), Amp (ampicillin resistance gene, comprising the promoter and ORF) and pUC ori (high-copy-number pUC origin of replication). (**B**) Western immunoblotting of pLV-CMV-(S-ΔRS-HA)-IRES-Puro-WPRE transfected HEK293T cell protein extracts (S-ΔRS-HA; 40 and 20 µg) and pEGFP-C1 transfected HEK293T cell protein extract (GFP; 40 µg) employed as a negative control (-). Cross-reactive bands in both lanes indicate the loading. (**C**) Representative microscopic image (phase contrast, fluorescence, and merged fields; 10X) of syncytia, generated by cotransfection of HEK/ACE2/TMPRRS2/Puro cells with pLV-CMV-(S-ΔRS-HA)-IRES-Puro-WPRE construct and PEGFP-C1. (**D**) Representative microscopic image (phase contrast, fluorescence, and merged fields; 10X) of syncytia, generated by cocultivation of HEK/ACE2/TMPRRS2/Puro cells with pLV-CMV-(S-ΔRS-HA)-IRES-Puro-WPRE and pEGFP-C1 cotransiently transfected HEK293T cells.

CoV-2 S ORF sequence (https://www.ncbi.nlm.nih.gov/protein/1791269090) was deprived of its last 57 bp, coding for a predicted ERRS (KFDEDDSEPVLKGVKLHYT) [14] and substituted with the hemagglutinin (HA) tag. The so-designed ORF (S-ΔRS-HA) was human codon usage adapted, with the use of the Jcat codon adaptation tool (http://www.jcat.de), to change nucleotide codon composition to a composition based on human genome codon usage. The degeneracy of the genetic code leads to a situation whereby most of the amino acids can be encoded by two to six synonymous codons. The synonymous codons are not equally utilized to encode the amino acids, thus resulting in the phenomenon of codon usage bias. As codon usage bias has been shown to correlate with gene expression level, it has been proposed as an important design parameter for enhancing recombinant protein production in heterologous host expression [17]. The GC content of S-ΔRS-HA is 37%, and adaptation to the human genome codon usage shifted the GC content from 37% up to 63% (Figure S2). Although we did not compare the adapted S-ΔRS-HA ORF with the unadapted one in terms of expression efficiency, previous studies have shown that GC-rich genes in mammalian cells can be expressed 100-fold more efficiently than their GC-poor counterparts due to increased steady-state mRNA levels [18]. S-ΔRS-HA was chemically synthesized and integrated into a transfer vector under the transcriptional guide of the immediate early gene promoter of human cytomegalovirus (CMV) and followed by an IRES, the puromycin drug resistance gene, and the woodchuck hepatitis virus (WHP) posttranscriptional regulatory element (WPRE) (pLV-CMV-(S-ΔRS-HA)-IRES-Puro-WPRE) (Figure 1A and Figure S3A for complete sequence).

pLV-CMV-(S-ΔRS-HA)-IRES-Puro-WPRE transfected cells successfully expressed S-ΔRS-HA protein, as shown by Western immunoblotting (Figure 1B). When HEK/ACE2/TMPRRS2/Puro were transiently cotransfected with pLV-CMV-(S-ΔRS-HA)-IRES-Puro-WPRE and pEGFP-C1, a construct delivering enhanced green fluorescent protein (EGFP), large syncytia were observed (Figure 1C). This syncytiogenic effect was attributable to the interaction between S, ACE2, and TMPRRS2, which recapitulated the bona fide fusogenic activity of S when it is attached to ACE2, proteolytically activated by TMPRSS2, and regulated by interferon-induced transmembrane proteins (IFITMs) [19,20]. An identical result was obtained when HEK/ACE2/TMPRRS2/Puro cells were cocultivated with pLV-CMV-(S-ΔRS-HA)-IRES-Puro-WPRE and pEGFP-C1 cotransiently transfected HEK293T cells (Figure 1D). Further, syncytia could be reduced or abrogated if pLV-CMV-(S-ΔRS-HA)-IRES-Puro-WPRE transfected HEK/ACE2/TMPRRS2/Puro cells were treated with human COVID-19 convalescent sera containing anti-S antibodies (Figure S3B).

### 3.3. Generation of a Lentivirus-Based Pseudovirus

**Figure 2:**
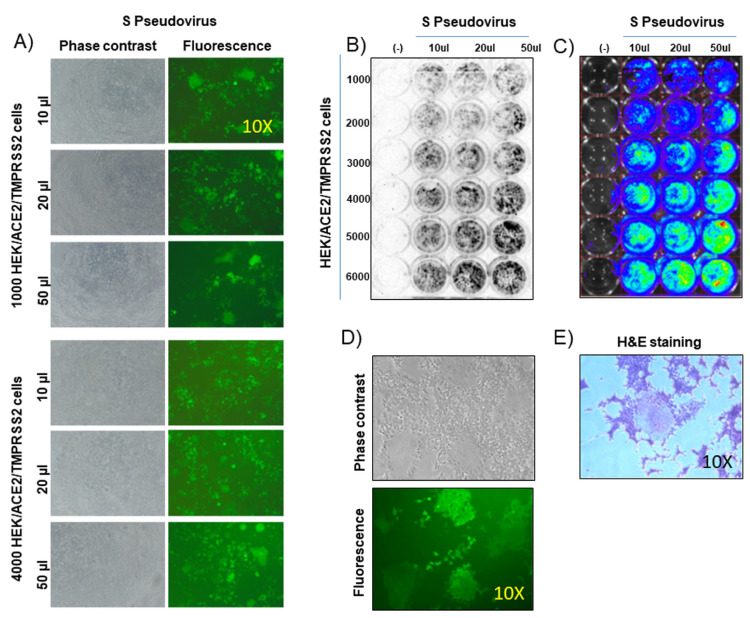
Pseudovirus assembly and transduction. (**A**) Representative images (phase contrast and fluorescence; 10X) of HEK/ACE2/TMPRRS2/Puro cells, at different numbers (1000 and 4000), transduced with different amounts (50, 20, and 10 µL) of supernatant containing pseudovirus. (**B**) ChemiDoc and (**C**) IVIS luminometric detection of HEK/ACE2/TMPRRS2/Puro cells, at different numbers (1000, 2000, 3000, 4000, 5000, and 6000), transduced with different amounts (50, 20, and 10 µL) of supernatant containing pseudovirus (S pseudovirus). Negative control was established with HEK293T cells, at different numbers (1000, 2000, 3000, 4000, 5000, and 6000) and transduced with 50 µL of the same supernatant containing pseudovirus (S pseudovirus). (**D**) Representative images of syncytia (phase contrast and fluorescence of the same field; 10X), generated by pseudovirus-transduced HEK/ACE2/TMPRRS2/Puro cells. (**E**) Representative images of a syncytium (phase contrast; 10X) generated by pseudovirus-transduced HEK/ACE2/TMPRRS2/Puro cells and stained with hematoxylin and eosin (H&E staining).

Relying on the promising results obtained with pLV-CMV-(S-ΔRS-HA)-IRES-Puro-WPRE construct and HEK/ACE2/TMPRRS2/Puro cell line, the reconstitution of a second-generation replicating incompetent lentivirus pseudotyped with CoV-2 S was attempted. The pLV-EF1α-(turboGFP-Luc2)-WPRE transfer vector, the p8.74 packaging vector, and the pLV-CMV-(S-ΔRS-HA)-IRES-Puro-WPRE pseudotyping vector were cotransfected in HEK293T cells. Twenty-four and forty-eight hours after transfection, transfected cell supernatant (TCS) was harvested, clarified, filtered, aliquoted, stored at −80 °C, and subsequently tested on HEK/ACE2/TMPRRS2/Puro cells and on HEK293T cells as negative control. As pLV-EF1α-(turboGFP-Luc2)-WPRE transfer vector could simultaneously deliver two reporter genes, turboGFP and a human codon usage adapted luciferase (Luc2), cell transduction could be detected either by fluorescence or luminometry. In fact, when different amounts of TCS were tested on different numbers of HEK/ACE2/TMPRRS2/Puro cells, efficient cell transduction could be observed, but not on HEK293T negative control cells (Figure 2A–C).

Further, bona fide fusogenic activity through syncytia formation could be observed (Figure 2D,E), recapitulating natural CoV-2 infection. Syncytia formation could be considered a signature of CoV infection and pathogenicity as it can be observed not only in vitro but also in vivo, in lung tissue from people infected with different CoVs, such as SARS-CoV, MERS-CoV, or SARS-CoV-2 [20,21,22,23,24,25]. 

### 3.4. Lentivirus-Based Vector Can Be Pseudotyped with ACE2/TMPRSS2

**Figure 3:**
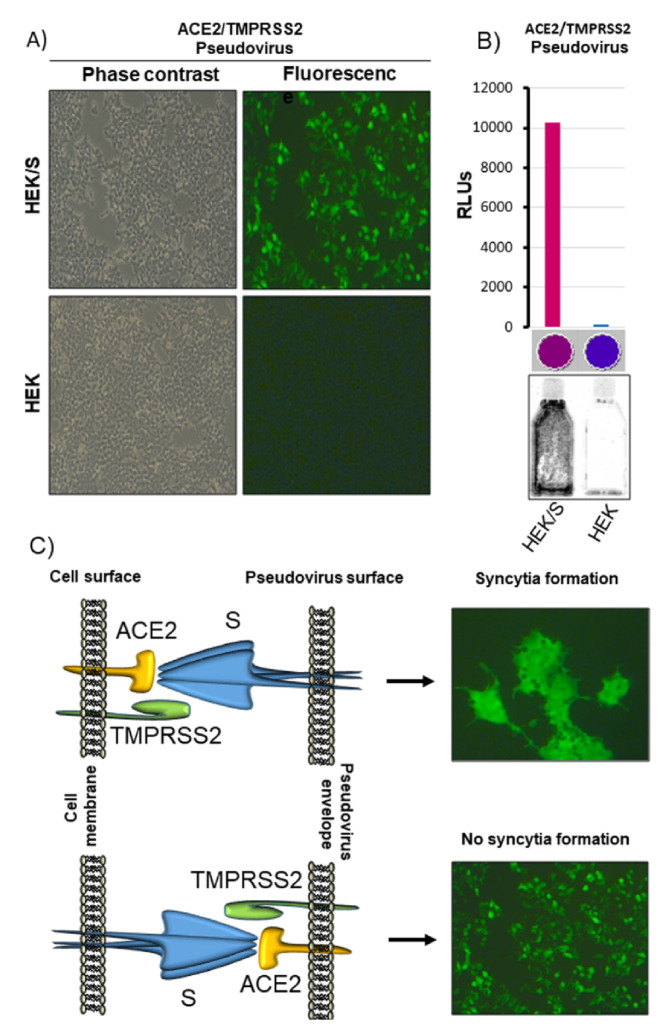
Pseudovirus generation with ACE2 and TMPRSS2. (**A**) Representative image (phase contrast and fluorescence of the same field; 10X) of HEK/S-ΔRS-HA/Puro cells (HEK/S) and HEK293T cells (HEK) transduced with ACE2/TMPRSS2 pseudovirus. (**B**) Representative 25 cm^2^ flasks of HEK/S-ΔRS-HA/Puro cells (HEK/S) and HEK293T cells (HEK) transduced with ACE2/TMPRSS2 pseudovirus and exposed to the ChemiDoc apparatus. Luminometric quantification of the lysate of the same cells, expressed as relative luciferase units (RLUs) and colorimetrically visualized (violet and blue spots) by the luminometer software, during and after reading. (**C**) Drawing explaining the lack of syncytia generation by ACE2/TMPRSS2 pseudovirus.

TMPRSS2 is a serine protease with a class II transmembrane domain, whereas ACE2 is a carboxypeptidase with a class I transmembrane domain, and both of them are localized on the cell membrane surface [8] (Figure S4). Based on this information, it was reasoned that lentiviral vector particles could potentially be pseudotyped with ACE2/TMPRSS2 and allow pseudovirus penetration into cells expressing CoV-2 S. The pLV-EF1α-(turboGFP-Luc2)-WPRE transfer vector, the p8.74 packaging vector, and the pEF1α-ACE2/TMPRRS2/Puro pseudotyping vector were cotransfected in HEK293T cells. Twenty-four and forty-eight hours after transfection, TCS was harvested, aliquoted, stored at −80 °C, and subsequently tested on pLV-CMV-(S-ΔRS-HA)-IRES-Puro-WPRE transiently or stably transfected HEK/S-ΔRS-HA/Puro cells and on HEK293T cells as negative control. TCS transduction could be observed on HEK/S-ΔRS-HA/Puro cells but not on HEK293T negative control cells (Figure 3A,B).

Therefore, a lentivirus-based vector could be pseudotyped with ACE2/TMPRSS2. Worthy of note, this kind of pseudotypization and cell transduction did not generate syncytia as observed for the lentivirus-based vector pseudotyped with CoV-2 S. Although the reason for this issue was not investigated, because such an investigation would be outside the scope of this work, it could be assumed that the syncytia formation is an active process in part dependent on a specific intracellular apparatus, contacting the long cytoplasmic tail of ACE2 and/or TMPRSS2, that is present inside the cells but absent inside the pseudotyped lentiviral particles. It was shown that infection with CoV-2 was significantly decreased in cells expressing ACE2 mutant versions lacking the cytoplasmic domain [26].

### 3.5. Assessment of a Pseudovirus Neutralization Assay

Starting from the availability of a pseudovirus mimicking the interaction of SARS-CoV-2 with target cells, it was of interest to assess a pseudovirus neutralization assay, to be applied for several purposes. The aim of this assay is to overcome the barriers presented by the complexities of other neutralization assays, such as multiple passages and the addition and removal of reagents and solutions, mainly during sera dilution and detection steps, where cells could detach from the microplate surface and serious errors could be introduced. In our simplified system, reagents and solution are only added at each step, while washing steps are absent, from the beginning to the end of the assay.

**Figure 4:**
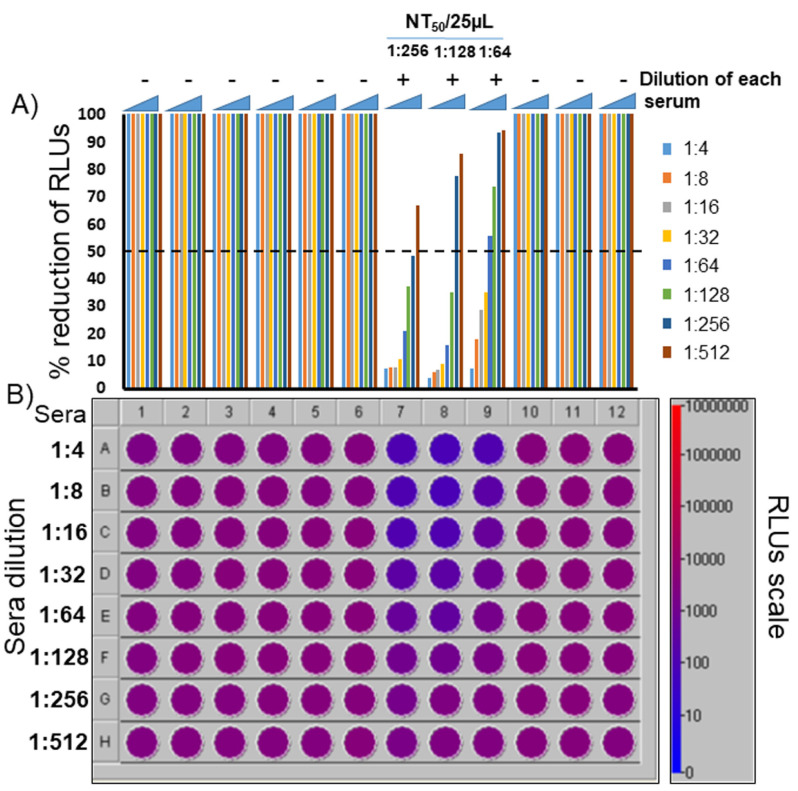
Representative pseudovirus neutralization assay. Six PCR and ELISA CoV-2 negative patients’ representative sera (1 to 6), three PCR and ELISA CoV-2 positive patients’ sera (7 to 9), and two pre-pandemic sera (collected in 1998; 10 and 11) were tested. A further negative control was established without serum (12), which was substituted with complete medium. (**A**) Histogram obtained from luminometric detection of transduced cells with pseudovirus. Relative luciferase units (RLUs) were compared and normalized to those derived from wells where pseudovirus was added in the absence of sera (100%). Neutralization titer 50 (NT_50_) was expressed as the maximal dilution of the sera where the reduction of the signal was ≥50%. In this specific example, the NT_50_/25 µL of serum 7 is 1:256, that of serum 8 is 1:128, and that of serum 9 is 1:64. However, because the titer was measured at 25 µL, the apparent NT_50_ has to be multiplied by 40 to obtain the normalized NT_50_/mL. Therefore, the NT_50_/mL of the sera 7 is 1:10,256, that of the serum 8 is 1:5120, and that of the serum 9 is 1:2560. Bar colors indicate sera dilution. (**B**) Colorimetric visualization of the RLUs displayed by the luminometer software, during and after reading.

Clear–flat-bottom 96-well microplates with opaque walls were employed (Figure S5). Opaque walls prevent well-to-well crosstalk for luminometric detection, whereas clear–flat-bottom wells allow direct microscopic monitoring of cell growth and/or fluorescence microscope observation of transduced cells expressing a fluorescent reporter gene, in this specific case turboGFP. The neutralization assay was performed with human sera coming from SARS-CoV-2-infected patients, collected after symptom onset, that produced positive results in PCR and ELISA tests. Sera collected prior to the SARS-CoV-2 emergence (1999) that tested negative in ELISA were used as negative control. First, serial dilutions of the sera (plasma from EDTA-treated blood can be used too) were incubated for 90 min with TCS containing pseudovirus sufficient to achieve approximately 10^4^ relative light units (RLUs) of luciferase signal per well (usually no more than 2 to 10 µL). Subsequently, 10^4^ HEK/ACE2/TMPRRS2/Puro cells were added to each well (Figure S5). As previously mentioned, HEK/ACE2/TMPRRS2/Puro cells do not need to be constantly kept in culture under puromycin selection: a large batch of them can be grown, aliquoted at a suitable concentration, and stored at −80 °C or in liquid nitrogen and be immediately ready to use after thawing. Alternatively, HEK293T cells can be electroporated with pEF1α-ACE2/TMPRRS2/Puro and directly used, obtaining the same result. NT50 could be measured as soon as 48 h after adding the cells, either by a fluorescence microscope or by luminometry and simply adding luciferin in each well (Figure S5 and Figure 4A,B), without the need for cell lysis and transferring the lysate to a different microplate. Moreover, light detection can be performed with a digital imager for Western blotting, BLI, or by a multiwell plate reader luminometer.

Thus, a rapid, flexible, reliable, sensitive, and cost-effective pseudovirus neutralization assay for SARS-CoV-2 was established, where all steps could be performed in the same plate and in a standard BSL2 laboratory, as the pseudovirus is completely safe. With this assay, we have been able to rapidly and cheaply determine the neutralizing potency of mAbs derived from serum or plasma samples in a BSL2 laboratory. Automation and additional miniaturization to further increase throughput are certainly possible. As serum neutralizing activity has long been identified as a correlate of protection in many viral infections [27], including CoV infections [28], and a global CoV-2 vaccination campaign is ongoing, it is absolutely important to have a practical test to assess the outcome of vaccination. In order to meet this need, such a test must be rapid, flexible, reliable, sensitive, quantitative, cost-effective, safe, and easily adapted to different coronavirus variants [29,30].

## Data Availability

Data is contained within the article. Reported results can be found in Supplementary Materials.

## Supplementary Materials

The following are available online at https://www.mdpi.com/article/10.3390/vaccines9040389/s1. Figure S1: ACE2/TMPRSS2 expressing construct.; Figure S2: S-ΔRS-HA human adapted ORF sequence and Biscistronic turboGFP-IRES-Luc2 ORF; Figure S3: pLV-CMV-(S-ΔRS-HA)-IRES-Puro-WPRE and representive phase contrast images (10X) of healthy donor serum treated pLV-CMV-(S-ΔRS-HA)-IRES-Puro-WPRE transfected HEK/ACE2/TMPRRS2/Puro cells (large syncytia formation) and COVID-19 convalescent serum treated pLV-CMV-(S-ΔRS-HA)-IRES-Puro-WPRE transfected HEK/ACE2/TMPRRS2/Puro cells (absence of syncytia).; Figure S4: ACE2 and TMPRSS2 topology; Figure S5: Serum Neutralization Flow-chart.
