# Supplementary material for: A Simplified SARS-CoV-2 Pseudovirus Neutralization Assay
Source: Vaccines (Basel). 2021 Apr 15;9(4):389. doi: 10.3390/vaccines9040389 (PMC8071407; doi:10.3390/vaccines9040389)
Supplement: Supplementary file 1 [file vaccines-09-00389-s001.pdf]

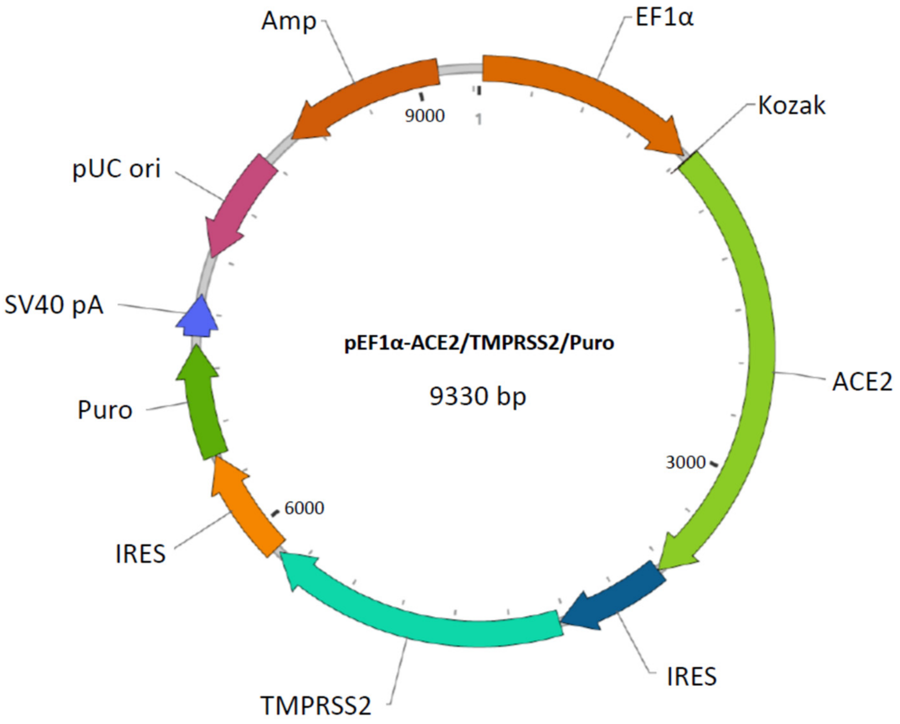

(A)

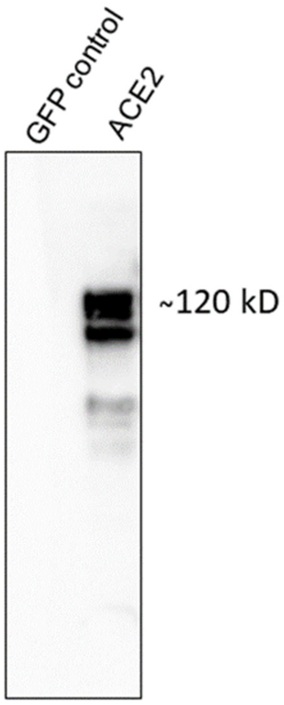

(B)

Figure S1. ACE2/TMPRSS2 expressing construct. (**A**) Diagram not on scale of pEF1α-ACE2/TMPRSS2/Puro construct, where functional elements of the construct are indicated and highlighted with different colours. EF1α (human Elongation Facto 1 alpha promoter), Kozak (Kozak's translation initiation sequence), ACE2 (Human Angiotensin Converting Enzyme 2 ORF), IRES (Encephalomyocarditis virus internal ribosomal entry site), TMPRSS2 (human Transmembrane Protease Serine 2), Puro (Puromycin resistant gene ORF), pUC ori (High copy number pUC origin of replication) SV40 early pA (Simian virus 40 early polyadenylation signal) and Amp (Ampicillin resistance gene, comprising the prokaryotic promoter and ORF). (**B**) Western immunoblotting of pEF1α-ACE2/TMPRSS2/Puro stably transfected HEK293T cells expressing ACE2. Negative control was established with HEK293T cell transfected with pEGFP-C1 plasmid. Each lane was loaded with 40 μg of cell protein extract.

pEF1A-ACE2/TMPRRS2/Puro complete sequence.

CAACTTTGTATAGAAAAGTTGGGCTCCGGTGCCCGTCAGTGGGCAGAGCGCACATCGCCC
ACAGTCCCCGAGAAGTTGGGGGGAGGGGTCGGCAATTGAACCGGTGCCTAGAGAAGGTGG
CGCGGGGTAAACTGGGAAAGTGATGTCGTGTACTGGCTCCGCCTTTTTCCCGAGGGTGGG
GGAGAACCGTATATAAGTGCAGTAGTCGCCGTGAACGTTCTTTTTCGCAACGGGTTTGCC
GCCAGAACACAGGTAAGTGCCGTGTGTGGTTCCCGCGGGCCTGGCCTCTTTACGGGTTAT
GGCCCTTGCGTGCCTTGAATTACTTCCACCTGGCTGCAGTACGTGATTCTTGATCCCGAG
CTTCGGGTTGGAAGTGGGTGGGAGAGTTCGAGGCCTTGCGCTTAAGGAGCCCCTTCGCCT
CGTGCTTGAGTTGAGGCCTGGCCTGGGCGCTGGGGCCGCCGCGTGCGAATCTGGTGGCAC
CTTCGCGCCTGTCTCGCTGCTTTCGATAAGTCTCTAGCCATTTAAAATTTTTGATGACCT
GCTGCGACGCTTTTTTTCTGGCAAGATAGTCTTGTAAATGCGGGCCAAGATCTGCACACT
GGTATTTCGGTTTTTGGGGCCGCGGGCGGCGACGGGGCCCGTGCGTCCCAGCGCACATGT
TCGGCGAGGCGGGGCCTGCGAGCGCGGCCACCGAGAATCGGACGGGGGTAGTCTCAAGCT
GGCCGGCCTGCTCTGGTGCCTGGTCTCGCGCCGCCGTGTATCGCCCCGCCCTGGGCGGCA
AGGCTGGCCCGGTCGGCACCAGTTGCGTGAGCGGAAAGATGGCCGCTTCCCGGCCCTGCT
GCAGGGAGCTCAAAATGGAGGACGCGGCGCTCGGGAGAGCGGGCGGGTGAGTCACCCACA
CAAAGGAAAAGGGCCTTTCCGTCCTCAGCCGTCGCTTCATGTGACTCCACGGAGTACCGG
GCGCCGTCCAGGCACCTCGATTAGTTCTCGAGCTTTTGGAGTACGTCGTCTTTAGGTTGG
GGGGAGGGGTTTTATGCGATGGAGTTTCCCCACACTGAGTGGGTGGAGACTGAAGTTAGG
CCAGCTTGGCACTTGATGTAATTCTCCTTGGAATTTGCCCTTTTTGAGTTTGGATCTTGG
TTCATTCTCAAGCCTCAGACAGTGGTTCAAAGTTTTTTTCTTCCATTTCAGGTGTCGTGA
CAAGTTTGTACAAAAAAGCAGGCTGCCACCATGTCAAGCTCTTCCTGGCTCCTTCTCAGC
CTTGTTGCTGTAACTGCTGCTCAGTCCACCATTGAGGAACAGGCCAAGACATTTTTGGAC
AAGTTTAACCACGAAGCCGAAGACCTGTTCTATCAAAGTTCACTTGCTTCTTGGAATTAT
AACACCAATATTACTGAAGAGAATGTCCAAAACATGAATAATGCTGGGGACAAATGGTCT
GCCTTTTTAAAGGAACAGTCCACACTTGCCCAAATGTATCCACTACAAGAAATTCAGAAT
CTCACAGTCAAGCTTCAGCTGCAGGCTCTTCAGCAAAATGGGTCTTCAGTGCTCTCAGAA
GACAAGAGCAAACGGTTGAACACAATTCTAAATACAATGAGCACCATCTACAGTACTGGA
AAAGTTTGTAACCCAGATAATCCACAAGAATGCTTATTACTTGAACCAGGTTTGAATGAA
ATAATGGCAAACAGTTTAGACTACAATGAGAGGCTCTGGGCTTGGGAAAGCTGGAGATCT
GAGGTCGGCAAGCAGCTGAGGCCATTATATGAAGAGTATGTGGTCTTGAAAAATGAGATG
GCAAGAGCAAATCATTATGAGGACTATGGGGATTATTGGAGAGGAGACTATGAAGTAAAT
GGGGTAGATGGCTATGACTACAGCCGCGGCCAGTTGATTGAAGATGTGGAACATACCTTT
GAAGAGATTAAACCATTATATGAACATCTTCATGCCTATGTGAGGGCAAAGTTGATGAAT
GCCTATCCTTCCTATATCAGTCCAATTGGATGCCTCCCTGCTCATTTGCTTGGTGATATG
TGGGGTAGATTTTGGACAAATCTGTACTCTTTGACAGTTCCCTTTGGACAGAAACCAAAC
ATAGATGTTACTGATGCAATGGTGGACCAGGCCTGGGATGCACAGAGAATATTCAAGGAG
GCCGAGAAGTTCTTTGTATCTGTTGGTCTTCCTAATATGACTCAAGGATTCTGGGAAAAT
TCCATGCTAACGGACCCAGGAAATGTTCAGAAAGCAGTCTGCCATCCCACAGCTTGGGAC
CTGGGGAAGGGCGACTTCAGGATCCTTATGTGCACAAAGGTGACAATGGACGACTTCCTG
ACAGCTCATCATGAGATGGGGCATATCCAGTATGATATGGCATATGCTGCACAACCTTTT
CTGCTAAGAAATGGAGCTAATGAAGGATTCCATGAAGCTGTTGGGGAAATCATGTCACTT
TCTGCAGCCACACCTAAGCATTTAAAATCCATTGGTCTTCTGTCACCCGATTTTCAAGAA
GACAATGAAACAGAAATAAACTTCCTGCTCAAACAAGCACTCACGATTGTTGGGACTCTG
CCATTTACTTACATGTTAGAGAAGTGGAGGTGGATGGTCTTTAAAGGGGAAATTCCCAAA
GACCAGTGGATGAAAAAGTGGTGGGAGATGAAGCGAGAGATAGTTGGGGTGGTGGAACCT
GTGCCCCATGATGAAACATACTGTGACCCCGCATCTCTGTTCCATGTTTCTAATGATTAC
TCATTCATTCGATATTACACAAGGACCCTTTACCAATTCCAGTTTCAAGAAGCACTTTGT
CAAGCAGCTAAACATGAAGGCCCTCTGCACAAATGTGACATCTCAAACTCTACAGAAGCT
GGACAGAAACTGTTCAATATGCTGAGGCTTGGAAAATCAGAACCCTGGACCCTAGCATTG
GAAAATGTTGTAGGAGCAAAGAACATGAATGTAAGGCCACTGCTCAACTACTTTGAGCCC
TTATTTACCTGGCTGAAAGACCAGAACAAGAATTCTTTTGTGGGATGGAGTACCGACTGG
AGTCCATATGCAGACCAAAGCATCAAAGTGAGGATAAGCCTAAAATCAGCTCTTGGAGAT
AAAGCATATGAATGGAACGACAATGAAATGTACCTGTTCCGATCATCTGTTGCATATGCT
ATGAGGCAGTACTTTTTAAAAGTAAAAAATCAGATGATTCTTTTTGGGGAGGAGGATGTG
CGAGTGGCTAATTTGAAACCAAGAATCTCCTTTAATTTCTTTGTCACTGCACCTAAAAAT
GTGTCTGATATCATTCCTAGAACTGAAGTTGAAAAGGCCATCAGGATGTCCCGGAGCCGT
ATCAATGATGCTTTCCGTCTGAATGACAACAGCCTAGAGTTTCTGGGGATACAGCCAACA
CTTGGACCTCCTAACCAGCCCCCTGTTTCCATATGGCTGATTGTTTTTGGAGTTGTGATG
GGAGTGATAGTGGTTGGCATTGTCATCCTGATCTTCACTGGGATCAGAGATCGGAAGAAG
AAAAATAAAGCAAGAAGTGGAGAAAATCCTTATGCCTCCATCGATATTAGCAAAGGAGAA
AATAATCCAGGATTCCAAAACACTGATGATGTTCAGACCTCCTTTTAGGCCCCTCTCCCT

CCCCCCCCCCTAACGTTACTGGCCGAAGCCGCTTGGAATAAGGCCGGTGTGCGTTTGTCT
ATATGTTATTTTCCACCATATTGCCGTCTTTTGGCAATGTGAGGGCCCGGAAACCTGGCC
CTGTCTTCTTGACGAGCATTCCTAGGGGTCTTTCCCCTCTCGCCAAAGGAATGCAAGGTC
TGTTGAATGTCGTGAAGGAAGCAGTTCCTCTGGAAGCTTCTTGAAGACAAACAACGTCTG
TAGCGACCCTTTGCAGGCAGCGGAACCCCCCACCTGGCGACAGGTGCCTCTGCGGCCAAA
AGCCACGTGTATAAGATACACCTGCAAAGGCGGCACAACCCCAGTGCCACGTTGTGAGTT
GGATAGTTGTGGAAAGAGTCAAATGGCTCTCCTCAAGCGTATTCAACAAGGGGCTGAAGG
ATGCCCAGAAGGTACCCCATTGTATGGGATCTGATCTGGGGCCTCGGTGCACATGCTTTA
CATGTGTTTAGTCGAGGTTAAAAAAACGTCTAGGCCCCCCGAACCACGGGGACGTGGTTT
TCCTTTGAAAAACACGATGATAATATGGCCACAACCATGCCCCCTGCCCCGCCCGGAGGT
GAAAGCGGGTGTGAGGAGCGCGGCGCGGCAGGTCATATTGAACATTCCAGATACCTATCA
TTACTCGATGCTGTTGATAACAGCAAGATGGCTTTGAACTCAGGGTCACCACCAGCTATT
GGACCTTACTATGAAAACCATGGATACCAACCGGAAAACCCCTATCCCGCACAGCCCACT
GTGGTCCCCACTGTCTACGAGGTGCATCCGGCTCAGTACTACCCGTCCCCCGTGCCCCAG
TACGCCCCGAGGGTCCTGACGCAGGCTTCCAACCCCGTCGTCTGCACGCAGCCCAAATCC
CCATCCGGGACAGTGTGCACCTCAAAGACTAAGAAAGCACTGTGCATCACCTTGACCCTG
GGGACCTTCCTCGTGGGAGCTGCGCTGGCCGCTGGCCTACTCTGGAAGTTCATGGGCAGC
AAGTGCTCCAACTCTGGGATAGAGTGCGACTCCTCAGGTACCTGCATCAACCCCTCTAAC
TGGTGTGATGGCGTGTCACACTGCCCCGGCGGGGAGGACGAGAATCGGTGTGTTCGCCTC
TACGGACCAAACTTCATCCTTCAGGTGTACTCATCTCAGAGGAAGTCCTGGCACCCTGTG
TGCCAAGACGACTGGAACGAGAACTACGGGCGGGCGGCCTGCAGGGACATGGGCTATAAG
AATAATTTTTACTCTAGCCAAGGAATAGTGGATGACAGCGGATCCACCAGCTTTATGAAA
CTGAACACAAGTGCCGGCAATGTCGATATCTATAAAAAACTGTACCACAGTGATGCCTGT
TCTTCAAAAGCAGTGGTTTCTTTACGCTGTATAGCCTGCGGGGTCAACTTGAACTCAAGC
CGCCAGAGCAGGATTGTGGGCGGCGAGAGCGCGCTCCCGGGGGCCTGGCCCTGGCAGGTC
AGCCTGCACGTCCAGAACGTCCACGTGTGCGGAGGCTCCATCATCACCCCCGAGTGGATC
GTGACAGCCGCCCACTGCGTGGAAAAACCTCTTAACAATCCATGGCATTGGACGGCATTT
GCGGGGATTTTGAGACAATCTTTCATGTTCTATGGAGCCGGATACCAAGTAGAAAAAGTG
ATTTCTCATCCAAATTATGACTCCAAGACCAAGAACAATGACATTGCGCTGATGAAGCTG
CAGAAGCCTCTGACTTTCAACGACCTAGTGAAACCAGTGTGTCTGCCCAACCCAGGCATG
ATGCTGCAGCCAGAACAGCTCTGCTGGATTTCCGGGTGGGGGGCCACCGAGGAGAAAGGG
AAGACCTCAGAAGTGCTGAACGCTGCCAAGGTGCTTCTCATTGAGACACAGAGATGCAAC
AGCAGATATGTCTATGACAACCTGATCACACCAGCCATGATCTGTGCCGGCTTCCTGCAG
GGGAACGTCGATTCTTGCCAGGGTGACAGTGGAGGGCCTCTGGTCACTTCGAAGAACAAT
ATCTGGTGGCTGATAGGGGATACAAGCTGGGGTTCTGGCTGTGCCAAAGCTTACAGACCA
GGAGTGTACGGGAATGTGATGGTATTCACGGACTGGATTTATCGACAAATGAGGGCAGAC
GGCTAAACCCAGCTTTCTTGTACAAAGTGGGCCCCTCTCCCTCCCCCCCCCCTAACGTTA
CTGGCCGAAGCCGCTTGGAATAAGGCCGGTGTGCGTTTGTCTATATGTTATTTTCCACCA
TATTGCCGTCTTTTGGCAATGTGAGGGCCCGGAAACCTGGCCCTGTCTTCTTGACGAGCA
TTCCTAGGGGTCTTTCCCCTCTCGCCAAAGGAATGCAAGGTCTGTTGAATGTCGTGAAGG
AAGCAGTTCCTCTGGAAGCTTCTTGAAGACAAACAACGTCTGTAGCGACCCTTTGCAGGC
AGCGGAACCCCCCACCTGGCGACAGGTGCCTCTGCGGCCAAAAGCCACGTGTATAAGATA
CACCTGCAAAGGCGGCACAACCCCAGTGCCACGTTGTGAGTTGGATAGTTGTGGAAAGAG
TCAAATGGCTCTCCTCAAGCGTATTCAACAAGGGGCTGAAGGATGCCCAGAAGGTACCCC
ATTGTATGGGATCTGATCTGGGGCCTCGGTGCACATGCTTTACATGTGTTTAGTCGAGGT
TAAAAAAACGTCTAGGCCCCCCGAACCACGGGGACGTGGTTTTCCTTTGAAAAACACGAT
GATAATATGGCCACAACCATGACCGAGTACAAGCCCACGGTGCGCCTCGCCACCCGCGAC
GACGTCCCCAGGGCCGTACGCACCCTCGCCGCCGCGTTCGCCGACTACCCCGCCACGCGC
CACACCGTCGATCCGGACCGCCACATCGAGCGGGTCACCGAGCTGCAAGAACTCTTCCTC
ACGCGCGTCGGGCTCGACATCGGCAAGGTGTGGGTCGCGGACGACGGCGCCGCGGTGGCG
GTCTGGACCACGCCGGAGAGCGTCGAAGCGGGGGCGGTGTTCGCCGAGATCGGCCCGCGC
ATGGCCGAGTTGAGCGGTTCCCGGCTGGCCGCGCAGCAACAGATGGAAGGCCTCCTGGCG
CCGCACCGGCCCAAGGAGCCCGCGTGGTTCCTGGCCACCGTCGGCGTCTCGCCCGACCAC
CAGGGCAAGGGTCTGGGCAGCGCCGTCGTGCTCCCCGGAGTGGAGGCGGCCGAGCGCGCC
GGGGTGCCCGCCTTCCTGGAGACCTCCGCGCCCCGCAACCTCCCCTTCTACGAGCGGCTC
GGCTTCACCGTCACCGCCGACGTCGAGGTGCCCGAAGGACCGCGCACCTGGTGCATGACC
CGCAAGCCCGGTGCCTGACAACTTTATTATACATAGTTGATGGCCGGCCGCTTCGAGCAG
ACATGATAAGATACATTGATGAGTTTGGACAAACCACAACTAGAATGCAGTGAAAAAAAT
GCTTTATTTGTGAAATTTGTGATGCTATTGCTTTATTTGTAACCATTATAAGCTGCAATA
AACAAGTTAACAACAACAATTGCATTCATTTTATGTTTCAGGTTCAGGGGGAGGTGTGGG
AGGTTTTTTAAAGCAAGTAAAACCTCTACAAATGTGGTAGCGGCCGCGGCGCTCTTCCGC
TTCCTCGCTCACTGACTCGCTGCGCTCGGTCGTTCGGCTGCGGCGAGCGGTATCAGCTCA
CTCAAAGGCGGTAATACGGTTATCCACAGAATCAGGGGATAACGCAGGAAAGAACATGTG

AGCAAAAGGCCAGCAAAAGGCCAGGAACCGTAAAAAGGCCGCGTTGCTGGCGTTTTTCCA
TAGGCTCCGCCCCCCTGACGAGCATCACAAAAATCGACGCTCAAGTCAGAGGTGGCGAAA
CCCGACAGGACTATAAAGATACCAGGCGTTTCCCCCTGGAAGCTCCCTCGTGCGCTCTCC
TGTTCCGACCCTGCCGCTTACCGGATACCTGTCCGCCTTTCTCTCTTCGGGAAGCGTGGC
GCTTTCTCATAGCTCACGCTGTAGGTATCTCAGTTCGGTGTAGGTCGTTCGCTCCAAGCT
GGGCTGTGTGCACGAACCCCCCGTTCAGCCCGACCGCTGCGCCTTATCCGGTAACTATCG
TCTTGAGTCCAACCCGGTAAGACACGACTTATCGCCACTGGCAGCAGCCACTGGTAACAG
GATTAGCAGAGCGAGGTATGTAGGCGGTGCTACAGAGTTCTTGAAGTGGTGGCCTAACTA
CGGCTACACTAGAAGAACAGTATTTGGTATCTGCGCTCTGCTGAAGCCAGTTACCTTCGG
AAAAAGAGTTGGTAGCTCTTGATCCGGCAAACAAACCACCGCTGGTAGCGGTGGTTTTTT
TGTTTGCAAGCAGCAGATTACGCGCAGAAAAAAAGGATCTCAAGAAGATCCTTTGATCTT
TTCTACGGGGTCTGACGCTCAGTGGAACGAAAACTCACGTTAAGGGATTTTGGTCATGAG
ATTATCAAAAAGGATCTTCACCTAGATCCTTTTAAATTAAAAATGAAGTTTTAAATCAAT
CTAAAGTATATATGAGTAAACTTGGTCTGACAGTTACCAATGCTTAATCAGTGAGGCACC
TATCTCAGCGATCTGTCTATTTCGTTCATCCATAGTTGCCTGACTCCCCGTCGTGTAGAT
AACTACGATACGGGAGGGCTTACCATCTGGCCCCAGTGCTGCAATGATACCGCGAGACCC
ACGCTCACCGGCTCCAGATTTATCAGCAATAAACCAGCCAGCCGGAAGGGCCGAGCGCAG
AAGTGGTCCTGCAACTTTATCCGCCTCCATCCAGTCTATTAATTGTTGCCGGGAAGCTAG
AGTAAGTAGTTCGCCAGTTAATAGTTTGCGCAACGTTGTTGCCATTGCTACAGGCATCGT
GGTGTCACGCTCGTCGTTTGGTATGGCTTCATTCAGCTCCGGTTCCCAACGATCAAGGCG
AGTTACATGATCCCCCATGTTGTGCAAAAAAGCGGTTAGCTCCTTCGGTCCTCCGATCGT
TGTCAGAAGTAAGTTGGCCGCAGTGTTATCACTCATGGTTATGGCAGCACTGCATAATTC
TCTTACTGTCATGCCATCCGTAAGATGCTTTTCTGTGACTGGTGAGTACTCAACCAAGTC
ATTCTGAGAATAGTGTATGCGGCGACCGAGTTGCTCTTGCCCGGCGTCAATACGGGATAA
TACCGCGCCACATAGCAGAACTTTAAAAGTGCTCATCATTGGAAAACGTTCTTCGGGGCG
AAAACTCTCAAGGATCTTACCGCTGTTGAGATCCAGTTCGATGTAACCCACTCGTGCACC
CAACTGATCTTCAGCATCTTTTACTTTCACCAGCGTTTCTGGGTGAGCAAAAACAGGAAG
GCAAAATGCCGCAAAAAAGGGAATAAGGGCGACACGGAAATGTTGAATACTCATACTCTT
CCTTTTTCAATATTATTGAAGCATTTATCAGGGTTATTGTCTCATGAGCGGATACATATT
TGAATGTATTTAGAAAAATAAACAAATAGGGGTTCCGCGCACATTTCCCCGAAAAGTGCC
ACCTGACGTCTAAGAAACCATTATTATCATGACATTAACCTATAAAAATAGGCGTATCAC
GAGGCCCTTTCGTCGGCGCGCCGCGGCCGC

Figure S2. S-ΔRS-HA human adapted ORF sequence.

ATGTTCGTGTTCCTGGTGCTGCTGCCCCTGGTGAGCAGCCAGTGCGTGAACCTGACCACCCGCACCCAGCTGCCCCCCGCCTACACCAACAGCTTC

ACCCGCGGCGTGTACTACCCCGACAAGGTGTTCCGCAGCAGCGTGCTGCACAGCACCCAGGACCTGTTCCTGCCCTTCTTCAGCAACGTGACCTG

GTTCCACGCCATCCACGTGAGCGGCACCAACGGCACCAAGCGCTTCGACAACCCCGTGCTGCCCTTCAACGACGGCGTGTACTTCGCCAGCACCG

AGAAGAGCAACATCATCCGCGGCTGGATCTTCGGCACCACCCTGGACAGCAAGACCCAGAGCCTGCTGATCGTGAACAACGCCACCAACGTGGT

GATCAAGGTGTGCGAGTTCCAGTTCTGCAACGACCCCTTCCTGGGCGTGTACTACCACAAGAACAACAAGAGCTGGATGGAGAGCGAGTTCCGC

GTGTACAGCAGCGCCAACAACTGCACCTTCGAGTACGTGAGCCAGCCCTTCCTGATGGACCTGGAGGGCAAGCAGGGCAACTTCAAGAACCTGC

GCGAGTTCGTGTTCAAGAACATCGACGGCTACTTCAAGATCTACAGCAAGCACACCCCCATCAACCTGGTGCGCGACCTGCCCCAGGGCTTCAGC

GCCCTGGAGCCCCTGGTGGACCTGCCCATCGGCATCAACATCACCCGCTTCCAGACCCTGCTGGCCCTGCACCGCAGCTACCTGACCCCCGGCGAC

AGCAGCAGCGGCTGGACCGCCGGCGCCGCCGCCTACTACGTGGGCTACCTGCAGCCCCGCACCTTCCTGCTGAAGTACAACGAGAACGGCACCA

TCACCGACGCCGTGGACTGCGCCCTGGACCCCCTGAGCGAGACCAAGTGCACCCTGAAGAGCTTCACCGTGGAGAAGGGCATCTACCAGACCAG

CAACTTCCGCGTGCAGCCCACCGAGAGCATCGTGCGCTTCCCCAACATCACCAACCTGTGCCCCTTCGGCGAGGTGTTCAACGCCACCCGCTTCGC

CAGCGTGTACGCCTGGAACCGCAAGCGCATCAGCAACTGCGTGGCCGACTACAGCGTGCTGTACAACAGCGCCAGCTTCAGCACCTTCAAGTGCT

ACGGCGTGAGCCCCACCAAGCTGAACGACCTGTGCTTCACCAACGTGTACGCCGACAGCTTCGTGATCCGCGGCGACGAGGTGCGCCAGATCGC

CCCCGGCCAGACCGGCAAGATCGCCGACTACAACTACAAGCTGCCCGACGACTTCACCGGCTGCGTGATCGCCTGGAACAGCAACAACCTGGACA

GCAAGGTGGGCGGCAACTACAACTACCTGTACCGCCTGTTCCGCAAGAGCAACCTGAAGCCCTTCGAGCGCGACATCAGCACCGAGATCTACCAG

GCCGGCAGCACCCCCTGCAACGGCGTGGAGGGCTTCAACTGCTACTTCCCCCTGCAGAGCTACGGCTTCCAGCCCACCAACGGCGTGGGCTACCA

GCCCTACCGCGTGGTGGTGCTGAGCTTCGAGCTGCTGCACGCCCCCGCCACCGTGTGCGGCCCCAAGAAGAGCACCAACCTGGTGAAGAACAAG

TGCGTGAACTTCAACTTCAACGGCCTGACCGGCACCGGCGTGCTGACCGAGAGCAACAAGAAGTTCCTGCCCTTCCAGCAGTTCGGCCGCGACAT

CGCCGACACCACCGACGCCGTGCGCGACCCCCAGACCCTGGAGATCCTGGACATCACCCCCTGCAGCTTCGGCGGCGTGAGCGTGATCACCCCCG

GCACCAACACCAGCAACCAGGTGGCCGTGCTGTACCAGGACGTGAACTGCACCGAGGTGCCCGTGGCCATCCACGCCGACCAGCTGACCCCCAC

CTGGCGCGTGTACAGCACCGGCAGCAACGTGTTCCAGACCCGCGCCGGCTGCCTGATCGGCGCCGAGCACGTGAACAACAGCTACGAGTGCGAC

ATCCCCATCGGCGCCGGCATCTGCGCCAGCTACCAGACCCAGACCAACAGCCCCCGCCGCGCCCGCAGCGTGGCCAGCCAGAGCATCATCGCCTA

CACCATGAGCCTGGGCGCCGAGAACAGCGTGGCCTACAGCAACAACAGCATCGCCATCCCCACCAACTTCACCATCAGCGTGACCACCGAGATCC

TGCCCGTGAGCATGACCAAGACCAGCGTGGACTGCACCATGTACATCTGCGGCGACAGCACCGAGTGCAGCAACCTGCTGCTGCAGTACGGCAG

CTTCTGCACCCAGCTGAACCGCGCCCTGACCGGCATCGCCGTGGAGCAGGACAAGAACACCCAGGAGGTGTTCGCCCAGGTGAAGCAGATCTAC

AAGACCCCCCCCATCAAGGACTTCGGCGGCTTCAACTTCAGCCAGATCCTGCCCGACCCCAGCAAGCCCAGCAAGCGCAGCTTCATCGAGGACCT

GCTGTTCAACAAGGTGACCCTGGCCGACGCCGGCTTCATCAAGCAGTACGGCGACTGCCTGGGCGACATCGCCGCCCGCGACCTGATCTGCGCCC

AGAAGTTCAACGGCCTGACCGTGCTGCCCCCCCTGCTGACCGACGAGATGATCGCCCAGTACACCAGCGCCCTGCTGGCCGGCACCATCACCAGC

GGCTGGACCTTCGGCGCCGGCGCCGCCCTGCAGATCCCCTTCGCCATGCAGATGGCCTACCGCTTCAACGGCATCGGCGTGACCCAGAACGTGCT

GTACGAGAACCAGAAGCTGATCGCCAACCAGTTCAACAGCGCCATCGGCAAGATCCAGGACAGCCTGAGCAGCACCGCCAGCGCCCTGGGCAAG

CTGCAGGACGTGGTGAACCAGAACGCCCAGGCCCTGAACACCCTGGTGAAGCAGCTGAGCAGCAACTTCGGCGCCATCAGCAGCGTGCTGAACG

ACATCCTGAGCCGCCTGGACAAGGTGGAGGCCGAGGTGCAGATCGACCGCCTGATCACCGGCCGCCTGCAGAGCCTGCAGACCTACGTGACCCA

GCAGCTGATCCGCGCCGCCGAGATCCGCGCCAGCGCCAACCTGGCCGCCACCAAGATGAGCGAGTGCGTGCTGGGCCAGAGCAAGCGCGTGGA

CTTCTGCGGCAAGGGCTACCACCTGATGAGCTTCCCCCAGAGCGCCCCCCACGGCGTGGTGTTCCTGCACGTGACCTACGTGCCCGCCCAGGAGA

AGAACTTCACCACCGCCCCCGCCATCTGCCACGACGGCAAGGCCCACTTCCCCCGCGAGGGCGTGTTCGTGAGCAACGGCACCCACTGGTTCGTG

Biscistronic turboGFP-IRES-Luc2 ORF

ATGCCCGCCATGAAGATCGAGTGCCGCATCACCGGCACCCTGAACGGCGTGGAGTTCGAGCTGGTGGGCGGCGGAGAGGGCACCCCCGAGCAG

GGCCGCATGACCAACAAGATGAAGAGCACCAAAGGCGCCCTGACCTTCAGCCCCTACCTGCTGAGCCACGTGATGGGCTACGGCTTCTACCACTT

CGGCACCTACCCCAGCGGCTACGAGAACCCCTTCCTGCACGCCATCAACAACGGCGGCTACACCAACACCCGCATCGAGAAGTACGAGGACGGC

GGCGTGCTGCACGTGAGCTTCAGCTACCGCTACGAGGCCGGCCGCGTGATCGGCGACTTCAAGGTGGTGGGCACCGGCTTCCCCGAGGACAGCG

TGATCTTCACCGACAAGATCATCCGCAGCAACGCCACCGTGGAGCACCTGCACCCCATGGGCGATAACGTGCTGGTGGGCAGCTTCGCCCGCACC

TTCAGCCTGCGCGACGGCGGCTACTACAGCTTCGTGGTGGACAGCCACATGCACTTCAAGAGCGCCATCCACCCCAGCATCCTGCAGAACGGGGG

CCCCATGTTCGCCTTCCGCCGCGTGGAGGAGCTGCACAGCAACACCGAGCTGGGCATCGTGGAGTACCAGCACGCCTTCAAGACCCCCATCGCCT

TCGCCAGATCTCGAGCTCGATGAACCCAGCTTTCTTGTACAAAGTGGGCCCCTCTCCCTCCCCCCCCCCTAACGTTACTGGCCGAAGCCGCTTGGAA

TAAGGCCGGTGTGCGTTTGTCTATATGTTATTTTCCACCATATTGCCGTCTTTTGGCAATGTGAGGGCCCGGAAACCTGGCCCTGTCTTCTTGACGA

GCATTCCTAGGGGTCTTTCCCCTCTCGCCAAAGGAATGCAAGGTCTGTTGAATGTCGTGAAGGAAGCAGTTCCTCTGGAAGCTTCTTGAAGACAA

ACAACGTCTGTAGCGACCCTTTGCAGGCAGCGGAACCCCCCACCTGGCGACAGGTGCCTCTGCGGCCAAAAGCCACGTGTATAAGATACACCTGC

AAAGGCGGCACAACCCCAGTGCCACGTTGTGAGTTGGATAGTTGTGGAAAGAGTCAAATGGCTCTCCTCAAGCGTATTCAACAAGGGGCTGAAG

GATGCCCAGAAGGTACCCCATTGTATGGGATCTGATCTGGGGCCTCGGTGCACATGCTTTACATGTGTTTAGTCGAGGTTAAAAAAACGTCTAGG

CCCCCCGAACCACGGGGACGTGGTTTTCCTTTGAAAAACACGATGATAATATGGCCACAACCATGGAAGATGCCAAAAACATTAAGAAGGGCCCA

GCGCCATTCTACCCACTCGAAGACGGGACCGCCGGCGAGCAGCTGCACAAAGCCATGAAGCGCTACGCCCTGGTGCCCGGCACCATCGCCTTTAC

CGACGCACATATCGAGGTGGACATTACCTACGCCGAGTACTTCGAGATGAGCGTTCGGCTGGCAGAAGCTATGAAGCGCTATGGGCTGAATACA

AACCATCGGATCGTGGTGTGCAGCGAGAATAGCTTGCAGTTCTTCATGCCCGTGTTGGGTGCCCTGTTCATCGGTGTGGCTGTGGCCCCAGCTAA

CGACATCTACAACGAGCGCGAGCTGCTGAACAGCATGGGCATCAGCCAGCCCACCGTCGTATTCGTGAGCAAGAAAGGGCTGCAAAAGATCCTC

AACGTGCAAAAGAAGCTACCGATCATACAAAAGATCATCATCATGGATAGCAAGACCGACTACCAGGGCTTCCAAAGCATGTACACCTTCGTGAC

TTCCCATTTGCCACCCGGCTTCAACGAGTACGACTTCGTGCCCGAGAGCTTCGACCGGGACAAAACCATCGCCCTGATCATGAACAGTAGTGGCA

GTACCGGATTGCCCAAGGGCGTAGCCCTACCGCACCGCACCGCTTGTGTCCGATTCAGTCATGCCCGCGACCCCATCTTCGGCAACCAGATCATCC

CCGACACCGCTATCCTCAGCGTGGTGCCATTTCACCACGGCTTCGGCATGTTCACCACGCTGGGCTACTTGATCTGCGGCTTTCGGGTCGTGCTCA

TGTACCGCTTCGAGGAGGAGCTATTCTTGCGCAGCTTGCAAGACTATAAGATTCAATCTGCCCTGCTGGTGCCCACACTATTTAGCTTCTTCGCTAA

GAGCACTCTCATCGACAAGTACGACCTAAGCAACTTGCACGAGATCGCCAGCGGCGGGGCGCCGCTCAGCAAGGAGGTAGGTGAGGCCGTGGC

CAAACGCTTCCACCTACCAGGCATCCGCCAGGGCTACGGCCTGACAGAAACAACCAGCGCCATTCTGATCACCCCCGAAGGGGACGACAAGCCTG

GCGCAGTAGGCAAGGTGGTGCCCTTCTTCGAGGCTAAGGTGGTGGACTTGGACACCGGTAAGACACTGGGTGTGAACCAGCGCGGCGAGCTGT

GCGTCCGTGGCCCCATGATCATGAGCGGCTACGTTAACAACCCCGAGGCTACAAACGCTCTCATCGACAAGGACGGCTGGCTGCACAGCGGCGA

CATCGCCTACTGGGACGAGGACGAGCACTTCTTCATCGTGGACCGGCTGAAGAGCCTGATCAAATACAAGGGCTACCAGGTAGCCCCAGCCGAA

CTGGAGAGCATCCTGCTGCAACACCCCAACATCTTCGACGCCGGGGTCGCCGGCCTGCCCGACGACGATGCCGGCGAGCTGCCCGCCGCAGTCG

TCGTGCTGGAACACGGTAAAACCATGACCGAGAAGGAGATCGTGGACTATGTGGCCAGCCAGGTTACAACCGCCAAGAAGCTGCGCGGTGGTG

TTGTGTTCGTGGACGAGGTGCCTAAAGGACTGACCGGCAAGTTGGACGCCCGCAAGATCCGCGAGATTCTCATTAAGGCCAAGAAGGGCGGCAA

GATCGCCGTGTAA

Figure S3. (**A**) pLV-CMV-(S-ΔRS-HA)-IRES-Puro-WPRE complete sequence

AATGTAGTCTTATGCAATACTCTTGTAGTCTTGCAACATGGTAACGATGAGTTAGCAACA
TGCCTTACAAGGAGAGAAAAAGCACCGTGCATGCCGATTGGTGGAAGTAAGGTGGTACGA
TCGTGCCTTATTAGGAAGGCAACAGACGGGTCTGACATGGATTGGACGAACCACTGAATT
GCCGCATTGCAGAGATATTGTATTTAAGTGCCTAGCTCGATACATAAACGGGTCTCTCTG
GTTAGACCAGATCTGAGCCTGGGAGCTCTCTGGCTAACTAGGGAACCCACTGCTTAAGCC
TCAATAAAGCTTGCCTTGAGTGCTTCAAGTAGTGTGTGCCCGTCTGTTGTGTGACTCTGG
TAACTAGAGATCCCTCAGACCCTTTTAGTCAGTGTGGAAAATCTCTAGCAGTGGCGCCCG
AACAGGGACTTGAAAGCGAAAGGGAAACCAGAGGAGCTCTCTCGACGCAGGACTCGGCTT
GCTGAAGCGCGCACGGCAAGAGGCGAGGGGCGGCGACTGGTGAGTACGCCAAAAATTTTG
ACTAGCGGAGGCTAGAAGGAGAGAGATGGGTGCGAGAGCGTCAGTATTAAGCGGGGGAGA
ATTAGATCGCGATGGGAAAAAATTCGGTTAAGGCCAGGGGGAAAGAAAAAATATAAATTA
AAACATATAGTATGGGCAAGCAGGGAGCTAGAACGATTCGCAGTTAATCCTGGCCTGTTA
GAAACATCAGAAGGCTGTAGACAAATACTGGGACAGCTACAACCATCCCTTCAGACAGGA
TCAGAAGAACTTAGATCATTATATAATACAGTAGCAACCCTCTATTGTGTGCATCAAAGG
ATAGAGATAAAAGACACCAAGGAAGCTTTAGACAAGATAGAGGAAGAGCAAAACAAAAGT
AAGACCACCGCACAGCAAGCGGCCGCTGATCTTCAGACCTGGAGGAGGAGATATGAGGGA
CAATTGGAGAAGTGAATTATATAAATATAAAGTAGTAAAAATTGAACCATTAGGAGTAGC
ACCCACCAAGGCAAAGAGAAGAGTGGTGCAGAGAGAAAAAAGAGCAGTGGGAATAGGAGC
TTTGTTCCTTGGGTTCTTGGGAGCAGCAGGAAGCACTATGGGCGCAGCGTCAATGACGCT
GACGGTACAGGCCAGACAATTATTGTCTGGTATAGTGCAGCAGCAGAACAATTTGCTGAG
GGCTATTGAGGCGCAACAGCATCTGTTGCAACTCACAGTCTGGGGCATCAAGCAGCTCCA
GGCAAGAATCCTGGCTGTGGAAAGATACCTAAAGGATCAACAGCTCCTGGGGATTTGGGG
TTGCTCTGGAAAACTCATTTGCACCACTGCTGTGCCTTGGAATGCTAGTTGGAGTAATAA
ATCTCTGGAACAGATTTGGAATCACACGACCTGGATGGAGTGGGACAGAGAAATTAACAA
TTACACAAGCTTAATACACTCCTTAATTGAAGAATCGCAAAACCAGCAAGAAAAGAATGA
ACAAGAATTATTGGAATTAGATAAATGGGCAAGTTTGTGGAATTGGTTTAACATAACAAA
TTGGCTGTGGTATATAAAATTATTCATAATGATAGTAGGAGGCTTGGTAGGTTTAAGAAT
AGTTTTTGCTGTACTTTCTATAGTGAATAGAGTTAGGCAGGGATATTCACCATTATCGTT
TCAGACCCACCTCCCAACCCCGAGGGGACCCGACAGGCCCGAAGGAATAGAAGAAGAAGG
TGGAGAGAGAGACAGAGACAGATCCATTCGATTAGTGAACGGATCTCGACGGTATCGCTA
GCTTTTAAAAGAAAAGGGGGGATTGGGGGGTACAGTGCAGGGGAAAGAATAGTAGACATA
ATAGCAACAGACATACAAACTAAAGAATTACAAAAACAAATTACAAAAATTCAAAATTTT
ACTAGTATCAACTTTGTATAGAAAAGTTGTAGTTATTAATAGTAATCAATTACGGGGTCA
TTAGTTCATAGCCCATATATGGAGTTCCGCGTTACATAACTTACGGTAAATGGCCCGCCT
GGCTGACCGCCCAACGACCCCCGCCCATTGACGTCAATAATGACGTATGTTCCCATAGTA
ACGCCAATAGGGACTTTCCATTGACGTCAATGGGTGGAGTATTTACGGTAAACTGCCCAC
TTGGCAGTACATCAAGTGTATCATATGCCAAGTACGCCCCCTATTGACGTCAATGACGGT
AAATGGCCCGCCTGGCATTATGCCCAGTACATGACCTTATGGGACTTTCCTACTTGGCAG
TACATCTACGTATTAGTCATCGCTATTACCATGGTGATGCGGTTTTGGCAGTACATCAAT
GGGCGTGGATAGCGGTTTGACTCACGGGGATTTCCAAGTCTCCACCCCATTGACGTCAAT
GGGAGTTTGTTTTGGCACCAAAATCAACGGGACTTTCCAAAATGTCGTAACAACTCCGCC
CCATTGACGCAAATGGGCGGTAGGCGTGTACGGTGGGAGGTCTATATAAGCAGAGCTGGT
TTAGTGAACCGTCAGATCCAAGTTTGTACAAAAAAGCAGGCTGCCACCATGTTCGTGTTC
CTGGTGCTGCTGCCCCTGGTGAGCAGCCAGTGCGTGAACCTGACCACCCGCACCCAGCTG
CCCCCCGCCTACACCAACAGCTTCACCCGCGGCGTGTACTACCCCGACAAGGTGTTCCGC
AGCAGCGTGCTGCACAGCACCCAGGACCTGTTCCTGCCCTTCTTCAGCAACGTGACCTGG
TTCCACGCCATCCACGTGAGCGGCACCAACGGCACCAAGCGCTTCGACAACCCCGTGCTG
CCCTTCAACGACGGCGTGTACTTCGCCAGCACCGAGAAGAGCAACATCATCCGCGGCTGG
ATCTTCGGCACCACCCTGGACAGCAAGACCCAGAGCCTGCTGATCGTGAACAACGCCACC
AACGTGGTGATCAAGGTGTGCGAGTTCCAGTTCTGCAACGACCCCTTCCTGGGCGTGTAC
TACCACAAGAACAACAAGAGCTGGATGGAGAGCGAGTTCCGCGTGTACAGCAGCGCCAAC
AACTGCACCTTCGAGTACGTGAGCCAGCCCTTCCTGATGGACCTGGAGGGCAAGCAGGGC
AACTTCAAGAACCTGCGCGAGTTCGTGTTCAAGAACATCGACGGCTACTTCAAGATCTAC
AGCAAGCACACCCCCATCAACCTGGTGCGCGACCTGCCCCAGGGCTTCAGCGCCCTGGAG
CCCCTGGTGGACCTGCCCATCGGCATCAACATCACCCGCTTCCAGACCCTGCTGGCCCTG
CACCGCAGCTACCTGACCCCCGGCGACAGCAGCAGCGGCTGGACCGCCGGCGCCGCCGCC
TACTACGTGGGCTACCTGCAGCCCCGCACCTTCCTGCTGAAGTACAACGAGAACGGCACC
ATCACCGACGCCGTGGACTGCGCCCTGGACCCCCTGAGCGAGACCAAGTGCACCCTGAAG
AGCTTCACCGTGGAGAAGGGCATCTACCAGACCAGCAACTTCCGCGTGCAGCCCACCGAG
AGCATCGTGCGCTTCCCCAACATCACCAACCTGTGCCCCTTCGGCGAGGTGTTCAACGCC
ACCCGCTTCGCCAGCGTGTACGCCTGGAACCGCAAGCGCATCAGCAACTGCGTGGCCGAC

TACAGCGTGCTGTACAACAGCGCCAGCTTCAGCACCTTCAAGTGCTACGGCGTGAGCCCC
ACCAAGCTGAACGACCTGTGCTTCACCAACGTGTACGCCGACAGCTTCGTGATCCGCGGC
GACGAGGTGCGCCAGATCGCCCCCGGCCAGACCGGCAAGATCGCCGACTACAACTACAAG
CTGCCCGACGACTTCACCGGCTGCGTGATCGCCTGGAACAGCAACAACCTGGACAGCAAG
GTGGGCGGCAACTACAACTACCTGTACCGCCTGTTCCGCAAGAGCAACCTGAAGCCCTTC
GAGCGCGACATCAGCACCGAGATCTACCAGGCCGGCAGCACCCCCTGCAACGGCGTGGAG
GGCTTCAACTGCTACTTCCCCCTGCAGAGCTACGGCTTCCAGCCCACCAACGGCGTGGGC
TACCAGCCCTACCGCGTGGTGGTGCTGAGCTTCGAGCTGCTGCACGCCCCCGCCACCGTG
TGCGGCCCCAAGAAGAGCACCAACCTGGTGAAGAACAAGTGCGTGAACTTCAACTTCAAC
GGCCTGACCGGCACCGGCGTGCTGACCGAGAGCAACAAGAAGTTCCTGCCCTTCCAGCAG
TTCGGCCGCGACATCGCCGACACCACCGACGCCGTGCGCGACCCCCAGACCCTGGAGATC
CTGGACATCACCCCCTGCAGCTTCGGCGGCGTGAGCGTGATCACCCCCGGCACCAACACC
AGCAACCAGGTGGCCGTGCTGTACCAGGACGTGAACTGCACCGAGGTGCCCGTGGCCATC
CACGCCGACCAGCTGACCCCCACCTGGCGCGTGTACAGCACCGGCAGCAACGTGTTCCAG
ACCCGCGCCGGCTGCCTGATCGGCGCCGAGCACGTGAACAACAGCTACGAGTGCGACATC
CCCATCGGCGCCGGCATCTGCGCCAGCTACCAGACCCAGACCAACAGCCCCCGCCGCGCC
CGCAGCGTGGCCAGCCAGAGCATCATCGCCTACACCATGAGCCTGGGCGCCGAGAACAGC
GTGGCCTACAGCAACAACAGCATCGCCATCCCCACCAACTTCACCATCAGCGTGACCACC
GAGATCCTGCCCGTGAGCATGACCAAGACCAGCGTGGACTGCACCATGTACATCTGCGGC
GACAGCACCGAGTGCAGCAACCTGCTGCTGCAGTACGGCAGCTTCTGCACCCAGCTGAAC
CGCGCCCTGACCGGCATCGCCGTGGAGCAGGACAAGAACACCCAGGAGGTGTTCGCCCAG
GTGAAGCAGATCTACAAGACCCCCCCCATCAAGGACTTCGGCGGCTTCAACTTCAGCCAG
ATCCTGCCCGACCCCAGCAAGCCCAGCAAGCGCAGCTTCATCGAGGACCTGCTGTTCAAC
AAGGTGACCCTGGCCGACGCCGGCTTCATCAAGCAGTACGGCGACTGCCTGGGCGACATC
GCCGCCCGCGACCTGATCTGCGCCCAGAAGTTCAACGGCCTGACCGTGCTGCCCCCCCTG
CTGACCGACGAGATGATCGCCCAGTACACCAGCGCCCTGCTGGCCGGCACCATCACCAGC
GGCTGGACCTTCGGCGCCGGCGCCGCCCTGCAGATCCCCTTCGCCATGCAGATGGCCTAC
CGCTTCAACGGCATCGGCGTGACCCAGAACGTGCTGTACGAGAACCAGAAGCTGATCGCC
AACCAGTTCAACAGCGCCATCGGCAAGATCCAGGACAGCCTGAGCAGCACCGCCAGCGCC
CTGGGCAAGCTGCAGGACGTGGTGAACCAGAACGCCCAGGCCCTGAACACCCTGGTGAAG
CAGCTGAGCAGCAACTTCGGCGCCATCAGCAGCGTGCTGAACGACATCCTGAGCCGCCTG
GACAAGGTGGAGGCCGAGGTGCAGATCGACCGCCTGATCACCGGCCGCCTGCAGAGCCTG
CAGACCTACGTGACCCAGCAGCTGATCCGCGCCGCCGAGATCCGCGCCAGCGCCAACCTG
GCCGCCACCAAGATGAGCGAGTGCGTGCTGGGCCAGAGCAAGCGCGTGGACTTCTGCGGC
AAGGGCTACCACCTGATGAGCTTCCCCCAGAGCGCCCCCCACGGCGTGGTGTTCCTGCAC
GTGACCTACGTGCCCGCCCAGGAGAAGAACTTCACCACCGCCCCCGCCATCTGCCACGAC
GGCAAGGCCCACTTCCCCCGCGAGGGCGTGTTCGTGAGCAACGGCACCCACTGGTTCGTG
ACCCAGCGCAACTTCTACGAGCCCCAGATCATCACCACCGACAACACCTTCGTGAGCGGC
AACTGCGACGTGGTGATCGGCATCGTGAACAACACCGTGTACGACCCCCTGCAGCCCGAG
CTGGACAGCTTCAAGGAGGAGCTGGACAAGTACTTCAAGAACCACACCAGCCCCGACGTG
GACCTGGGCGACATCAGCGGCATCAACGCCAGCGTGGTGAACATCCAGAAGGAGATCGAC
CGCCTGAACGAGGTGGCCAAGAACCTGAACGAGAGCCTGATCGACCTGCAGGAGCTGGGC
AAGTACGAGCAGTACATCAAGTGGCCCTGGTACATCTGGCTGGGCTTCATCGCCGGCCTG
ATCGCCATCGTGATGGTGACCATCATGCTGTGCTGCATGACCAGCTGCTGCAGCTGCCTG
AAGGGCTGCTGCAGCTGCGGCAGCTGCTGCTACCCCTACGACGTGCCCGACTACGCCTAA
ACCCAGCTTTCTTGTACAAAGTGGCCCCTCTCCCTCCCCCCCCCCTAACGTTACTGGCC
GAAGCCGCTTGGAATAAGGCCGGTGTGCGTTTGTCTATATGTTATTTTCCACCATATTGC
CGTCTTTTGGCAATGTGAGGGCCCGGAAACCTGGCCCTGTCTTCTTGACGAGCATTCCTA
GGGGTCTTTCCCCTCTCGCCAAAGGAATGCAAGGTCTGTTGAATGTCGTGAAGGAAGCAG
TTCCTCTGGAAGCTTCTTGAAGACAAACAACGTCTGTAGCGACCCTTTGCAGGCAGCGGA
ACCCCCCACCTGGCGACAGGTGCCTCTGCGGCCAAAAGCCACGTGTATAAGATACACCTG
CAAAGGCGGCACAACCCCAGTGCCACGTTGTGAGTTGGATAGTTGTGGAAAGAGTCAAAT
GGCTCTCCTCAAGCGTATTCAACAAGGGGCTGAAGGATGCCCAGAAGGTACCCCATTGTA
TGGGATCTGATCTGGGGCCTCGGTGCACATGCTTTACATGTGTTTAGTCGAGGTTAAAAA
AACGTCTAGGCCCCCCGAACCACGGGGACGTGGTTTTCCTTTGAAAAACACGATGATAAT
ATGGCCACAACCATGACCGAGTACAAGCCCACGGTGCGCCTCGCCACCCGCGACGACGTC
CCCAGGGCCGTACGCACCCTCGCCGCCGCGTTCGCCGACTACCCCGCCACGCGCCACACC
GTCGATCCGGACCGCCACATCGAGCGGGTCACCGAGCTGCAAGAACTCTTCCTCACGCGC
GTCGGGCTCGACATCGGCAAGGTGTGGGTCGCGGACGACGGCGCCGCGGTGGCGGTCTGG
ACCACGCCGGAGAGCGTCGAAGCGGGGGCGGTGTTCGCCGAGATCGGCCCGCGCATGGCC
GAGTTGAGCGGTTCCCGGCTGGCCGCGCAGCAACAGATGGAAGGCCTCCTGGCGCCGCAC
CGGCCCAAGGAGCCCGCGTGGTTCCTGGCCACCGTCGGCGTCTCGCCCGACCACCAGGGC
AAGGGTCTGGGCAGCGCCGTCGTGCTCCCCGGAGTGGAGGCGGCCGAGCGCGCCGGGGTG

CCCGCCTTCCTGGAGACCTCCGCGCCCCGCAACCTCCCCTTCTACGAGCGGCTCGGCTTC
ACCGTCACCGCCGACGTCGAGGTGCCCGAAGGACCGCGCACCTGGTGCATGACCCGCAAG
CCCGGTGCCTGACAACTTTATTATACATAGTTGATCAATTCCGATAATCAACCTCTGGAT
TACAAAATTTGTGAAAGATTGACTGGTATTCTTAACTATGTTGCTCCTTTTACGCTATGT
GGATACGCTGCTTTAATGCCTTTGTATCATGCTATTGCTTCCCGTATGGCTTTCATTTTC
TCCTCCTTGTATAAATCCTGGTTGCTGTCTCTTTATGAGGAGTTGTGGCCCGTTGTCAGG
CAACGTGGCGTGGTGTGCACTGTGTTTGCTGACGCAACCCCCACTGGTTGGGGCATTGCC
ACCACCTGTCAGCTCCTTTCCGGGACTTTCGCTTTCCCCCTCCCTATTGCCACGGCGGAA
CTCATCGCCGCCTGCCTTGCCCGCTGCTGGACAGGGGCTCGGCTGTTGGGCACTGACAAT
TCCGTGGTGTTGTCGGGGAAGCTGACGTCCTTTCCATGGCTGCTCGCCTGTGTTGCCACC
TGGATTCTGCGCGGGACGTCCTTCTGCTACGTCCCTTCGGCCCTCAATCCAGCGGACCTT
CCTTCCCGCGGCCTGCTGCCGGCTCTGCGGCCTCTTCCGCGTCTTCGCCTTCGCCCTCAG
ACGAGTCGGATCTCCCTTTGGGCCGCCTCCCCGCATCGGGAATTCCCGCGGTTCGCTTTA
AGACCAATGACTTACAAGGCAGCTGTAGATCTTAGCCACTTTTTAAAAGAAAAGGGGGGA
CTGGAAGGGCTAATTCACTCCCAACGAAGACAAGATCTGCTTTTTGCTTGTACTGGGTCT
CTCTGGTTAGACCAGATCTGAGCCTGGGAGCTCTCTGGCTAACTAGGGAACCCACTGCTT
AAGCCTCAATAAAGCTTGCCTTGAGTGCTTCAAGTAGTGTGTGCCCGTCTGTTGTGTGAC
TCTGGTAACTAGAGATCCCTCAGACCCTTTTAGTCAGTGTGGAAAATCTCTAGCAGTAGT
AGTTCATGTCATCTTATTATTCAGTATTTATAACTTGCAAAGAAATGAATATCAGAGAGT
GAGAGGAACTTGTTTATTGCAGCTTATAATGGTTACAAATAAAGCAATAGCATCACAAAT
TTCACAAATAAAGCATTTTTTTCACTGCATTCTAGTTGTGGTTTGTCCAAACTCATCAAT
GTATCTTATCATGTCTGGCTCTAGCTATCCCGCCCCTAACTCCGCCCATCCCGCCCCTAA
CTCCGCCCAGTTCCGCCCATTCTCCGCCCCATGGCTGACTAATTTTTTTTATTTATGCAG
AGGCCGAGGCCGCCTCGGCCTCTGAGCTATTCCAGAAGTAGTGAGGAGGCTTTTTTGGAG
GCCTAGGGACGTACCCAATTCGCCCTATAGTGAGTCGTATTACGCGCGCTCACTGGCCGT
CGTTTTACAACGTCGTGACTGGGAAAACCCTGGCGTTACCCAACTTAATCGCCTTGCAGC
ACATCCCCCTTTCGCCAGCTGGCGTAATAGCGAAGAGGCCCGCACCGATCGCCCTTCCCA
ACAGTTGCGCAGCCTGAATGGCGAATGGGACGCGCCCTGTAGCGGCGCATTAAGCGCGGC
GGGTGTGGTGGTTACGCGCAGCGTGACCGCTACACTTGCCAGCGCCCTAGCGCCCGCTCC
TTTCGCTTTCTTCCCTTCCTTTCTCGCCACGTTCGCCGGCTTTCCCCGTCAAGCTCTAAA
TCGGGGGCTCCCTTTAGGGTTCCGATTTAGTGCTTTACGGCACCTCGACCCCAAAAAACT
TGATTAGGGTGATGGTTCACGTAGTGGGCCATCGCCCTGATAGACGGTTTTTCGCCCTTT
GACGTTGGAGTCCACGTTCTTTAATAGTGGACTCTTGTTCCAAACTGGAACAACACTCAA
CCCTATCTCGGTCTATTCTTTTGATTTATAAGGGATTTTGCCGATTTCGGCCTATTGGTT
AAAAAATGAGCTGATTTAACAAAAATTTAACGCGAATTTTAACAAAATATTAACGCTTAC
AATTTAGGTGGCACTTTTCGGGGAAATGTGCGCGGAACCCCTATTTGTTTATTTTTCTAA
ATACATTCAAATATGTATCCGCTCATGAGACAATAACCCTGATAAATGCTTCAATAATAT
TGAAAAAGGAAGAGTATGAGTATTCAACATTTCCGTGTCGCCCTTATTCCCTTTTTTGCG
GCATTTTGCCTTCCTGTTTTTGCTCACCCAGAAACGCTGGTGAAAGTAAAAGATGCTGAA
GATCAGTTGGGTGCACGAGTGGGTTACATCGAACTGGATCTCAACAGCGGTAAGATCCTT
GAGAGTTTTCGCCCCGAAGAACGTTTTCCAATGATGAGCACTTTTAAAGTTCTGCTATGT
GGCGCGGTATTATCCCGTATTGACGCCGGGCAAGAGCAACTCGGTCGCCGCATACACTAT
TCTCAGAATGACTTGGTTGAGTACTCACCAGTCACAGAAAAGCATCTTACGGATGGCATG
ACAGTAAGAGAATTATGCAGTGCTGCCATAACCATGAGTGATAACACTGCGGCCAACTTA
CTTCTGACAACGATCGGAGGACCGAAGGAGCTAACCGCTTTTTTGCACAACATGGGGGAT
CATGTAACTCGCCTTGATCGTTGGGAACCGGAGCTGAATGAAGCCATACCAAACGACGAG
CGTGACACCACGATGCCTGTAGCAATGGCAACAACGTTGCGCAAACTATTAACTGGCGAA
CTACTTACTCTAGCTTCCCGGCAACAATTAATAGACTGGATGGAGGCGGATAAAGTTGCA
GGACCACTTCTGCGCTCGGCCCTTCCGGCTGGCTGGTTTATTGCTGATAAATCTGGAGCC
GGTGAGCGTGGGTCTCGCGGTATCATTGCAGCACTGGGGCCAGATGGTAAGCCCTCCCGT
ATCGTAGTTATCTACACGACGGGGAGTCAGGCAACTATGGATGAACGAAATAGACAGATC
GCTGAGATAGGTGCCTCACTGATTAAGCATTGGTAACTGTCAGACCAAGTTTACTCATAT
ATACTTTAGATTGATTTAAAACTTCATTTTTAATTTAAAAGGATCTAGGTGAAGATCCTT
TTTGATAATCTCATGACCAAAATCCCTTAACGTGAGTTTTCGTTCCACTGAGCGTCAGAC
CCCGTAGAAAAGATCAAAGGATCTTCTTGAGATCCTTTTTTTCTGCGCGTAATCTGCTGC
TTGCAAACAAAAAAACCACCGCTACCAGCGGTGGTTTGTTTGCCGGATCAAGAGCTACCA
ACTCTTTTTCCGAAGGTAACTGGCTTCAGCAGAGCGCAGATACCAAATACTGTTCTTCTA
GTGTAGCCGTAGTTAGGCCACCACTTCAAGAACTCTGTAGCACCGCCTACATACCTCGCT
CTGCTAATCCTGTTACCAGTGGCTGCTGCCAGTGGCGATAAGTCGTGTCTTACCGGGTTG
GACTCAAGACGATAGTTACCGGATAAGGCGCAGCGGTCGGGCTGAACGGGGGGTTCGTGC
ACACAGCCCAGCTTGGAGCGAACGACCTACACCGAACTGAGATACCTACAGCGTGAGCTA
TGAGAAAGCGCCACGCTTCCCGAAGAGAGAAAGGCGGACAGGTATCCGGTAAGCGGCAGG
GTCGGAACAGGAGAGCGCACGAGGGAGCTTCCAGGGGGAAACGCCTGGTATCTTTATAGT

CCTGTCGGGTTTCGCCACCTCTGACTTGAGCGTCGATTTTTGTGATGCTCGTCAGGGGGG
CGGAGCCTATGGAAAAACGCCAGCAACGCGGCCTTTTTACGGTTCCTGGCCTTTTGCTGG
CCTTTTGCTCACATGTTCTTTCCTGCGTTATCCCCTGATTCTGTGGATAACCGTATTACC
GCCTTTGAGTGAGCTGATACCGCTCGCCGCAGCCGAACGACCGAGCGCAGCGAGTCAGTG
AGCGAGGAAGCGGAAGAGCGCCCAATACGCAAACCGCCTCTCCCCGCGCGTTGGCCGATT
CATTAATGCAGCTGGCACGACAGGTTTCCCGACTGGAAAGCGGGCAGTGAGCGCAACGCA
ATTAATGTGAGTTAGCTCACTCATTAGGCACCCCAGGCTTTACACTTTATGCTTCCGGCT
CGTATGTTGTGTGGAATTGTGAGCGGATAACAATTTCACACAGGAAACAGCTATGACCAT
GATTACGCCAAGCGCGCAATTAACCCTCACTAAAGGGAACAAAAGCTGGAGCTGCAAGCT
T

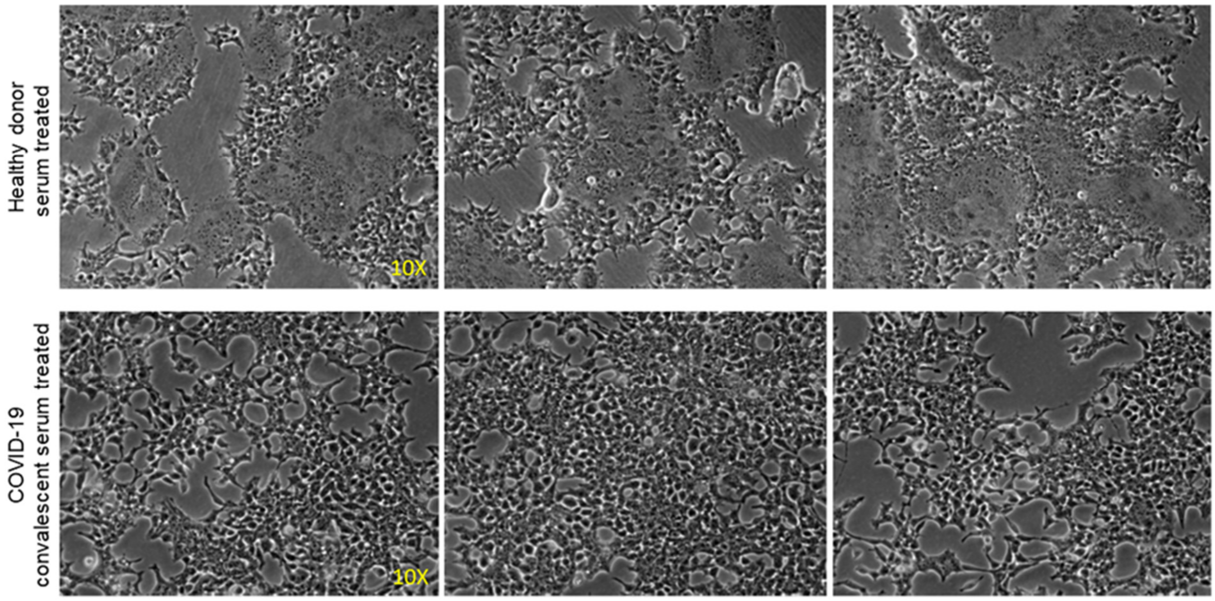

(**B**) Three representive phase contrast images (10X) of healthy donor serum treated pLV-CMV-(S-ΔRS-HA)-IRES-Puro-WPRE transfected HEK/ACE2/TMPRRS2/Puro cells (large syncytia formation) and COVID-19 convalescent serum treated pLV-CMV-(S-ΔRS-HA)-IRES-Puro-WPRE transfected HEK/ACE2/TMPRRS2/Puro cells (absence of syncytia). HEK/ACE2/TMPRRS2/Puro cells were treated with serum before and after transfection with pLV-CMV-(S-ΔRS-HA)-IRES-Puro-WPRE. The experiment was repeated 4 times with identical results

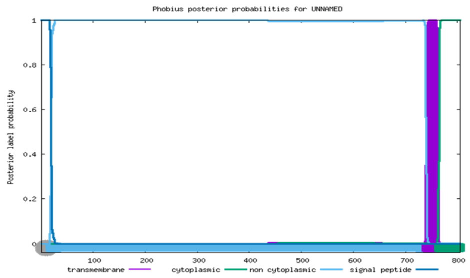

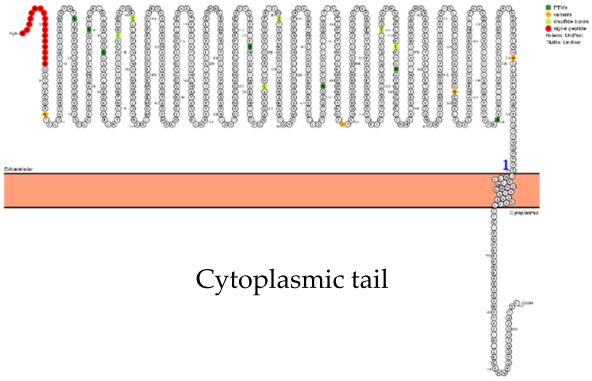

(A) **(B)**

ACE2 Type I TM

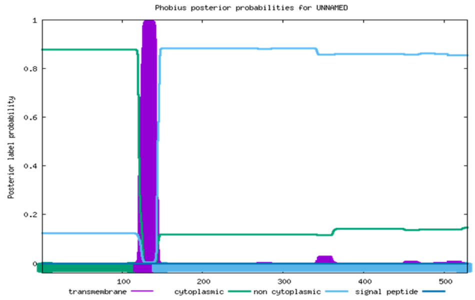

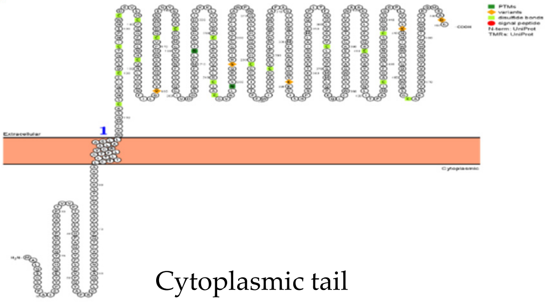

(A) **(B)**

TMPRSS2 Type II TM

Figure S4. ACE2 and TMPRSS2 topology: (**A**) A combined transmembrane topology and signal peptide prediction of ACE2 and TMPRSS2 as performed by Phobius (https://phobius.sbc.su.se). (**B**) A topological and subcellular localization prediction of ACE2 and TMPRSS2 as performed by Protter (https://wlab.ethz.ch/protter/start/). Open-source tool for visualization of proteoforms and interactive integration of annotated and predicted sequence features together with experimental proteomic evidence.

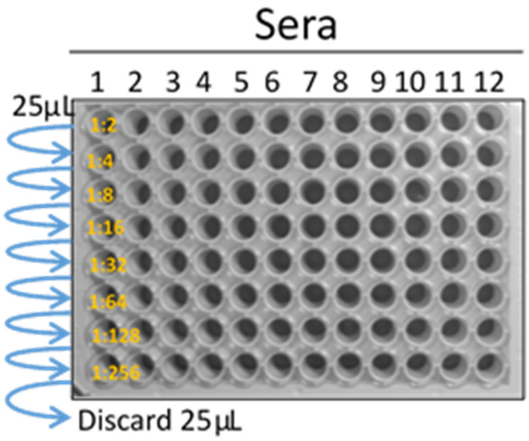

Figure S5. Serum Neutralization Flow-chart.

(1) Dilution of the sera

25µL of complete medium are added to each well of the microplate and 25 µL of each serum is added to each well of the first line of wells. Than 25 µL of the first dilution of the sera (1:2) are mixed and passed to the subsequent lines of wells. 25µL from the last line of wells are discarded. Therefore, the final volume for each well is 25µL.

(2) Adding the pseudovirus

25µL of pseudovirus in complete medium (corresponding to 104 RLUs; ~3-5µL of the initial preparation) are added to each well and left at room temperature for 1,5 hours. Final volume for each well is 50µL, therefore the sera dilution is doubled (1:4-1:8-1:16-1:32-1:64-1:128-1:256-1:512).

(3) Adding the cells

50µL of complete medium containing 10^4 HEK/ACE2/TMPRRS2/Puro cells are added to each well and left for 60 h at 37 °C and 5% CO_2. Final volume for each well is 100µL.

(4) Adding the Luciferine and reading

25µL of complete medium containing Luciferin (20µL of complete medium plus 5µL of 15mg/mL Luciferin in PBS) are added to each well just before the luminometric reading of the microplate. Final volume for each well is 125µL.

RLUs obtained were compared and normalized to those derived from wells where pseudovirus were added in the absence of plasma/sera (100%). Neutralization titer 50 (NT50/mL) was expressed as the maximal dilution of the sera where the reduction of the signal is ≥ 50%. This titer has to be multiplied per 40 because the initial volume of the sera tested is 0,025mL and it has to be normalized to 1mL. This is a very important issue because SN protocol could differ from each lab and the initial amount of sera tested could be different in volume. However, in most of the case, operators tend to indicate SN50 and not SN50/mL.
